# The RNA-binding protein ROD1/PTBP3 cotranscriptionally defines AID-loading sites to mediate antibody class switch in mammalian genomes

**DOI:** 10.1038/s41422-018-0076-9

**Published:** 2018-08-24

**Authors:** Juan Chen, Zhaokui Cai, Meizhu Bai, Xiaohua Yu, Chao Zhang, Changchang Cao, Xihao Hu, Lei Wang, Ruibao Su, Di Wang, Lei Wang, Yingpeng Yao, Rong Ye, Baidong Hou, Yang Yu, Shuyang Yu, Jinsong Li, Yuanchao Xue

**Affiliations:** 1 Key Laboratory of RNA Biology, CAS Center for Excellence in Biomacromolecules, Institute of Biophysics, Chinese Academy of Sciences, 100101 Beijing, China; 2 University of Chinese Academy of Sciences, 100049 Beijing, China; 3 State Key Laboratory of Molecular Biology, Shanghai Key Laboratory of Molecular Andrology, CAS Center for Excellence in Molecular Cell Science, Shanghai Institute of Biochemistry and Cell Biology, Chinese Academy of Sciences; University of Chinese Academy of Sciences, 320 Yueyang Road, 200031 Shanghai, China; 4 School of Life Science and Technology, ShanghaiTech University, 100 Haike Road, 201210 Shanghai, China; 5 Key Laboratory of Infection and Immunity, Institute of Biophysics, Chinese Academy of Sciences, 100101 Beijing, China; 6 College of Life Sciences, Institute for Conservation and Utilization of Agro-bioresources in Dabie Mountains, Xinyang Normal University, 464000 Xinyang, China; 7 State Key Laboratory of Agrobiotechnology, College of Biological Sciences, China Agricultural University, Beijing, People’s Republic of China

**Keywords:** Immunology, Non-coding RNAs

## Abstract

Activation-induced cytidine deaminase (AID) mediates class switching by binding to a small fraction of single-stranded DNA (ssDNA) to diversify the antibody repertoire. The precise mechanism for highly selective AID targeting in the genome has remained elusive. Here, we report an RNA-binding protein, ROD1 (also known as *PTBP3*), that is both required and sufficient to define AID-binding sites genome-wide in activated B cells. ROD1 interacts with AID via an ultraconserved loop, which proves to be critical for the recruitment of AID to ssDNA using bi-directionally transcribed nascent RNAs as stepping stones. Strikingly, AID-specific mutations identified in human patients with hyper-IgM syndrome type 2 (HIGM2) completely disrupt the AID interacting surface with ROD1, thereby abolishing the recruitment of AID to immunoglobulin (*Ig*) loci. Together, our results suggest that bi-directionally transcribed RNA traps the RNA-binding protein ROD1, which serves as a guiding system for AID to load onto specific genomic loci to induce DNA rearrangement during immune responses.

## Introduction

B cells produce a huge number of different antibodies to execute immune clearance of toxins, viruses and various microorganisms.^1^ Once activated in germinal centers, B cells express AID to initiate somatic hypermutation (SHM) in exons in variable regions, promoting affinity maturation.^2,3^ In the meantime, these cells also evoke class switch recombination (CSR) between the Cμ constant exon and one of the downstream exons, such as Cγ, Cα, or Cε, to produce high-affinity IgG, IgA, or IgE antibodies.^2,4,5^ AID, an enzyme that deaminates cytidines in single-stranded DNA (ssDNA) hotspots,^6–9^ is primarily loaded onto immunoglobulin (*Ig*) loci to induce double-strand breaks or mutations.^10,11^ Motifs enriched in AID hotspots are RGYW/WRCY (where R = purine, Y = pyrimidine, and W = A or T),^9^ which in theory appear once every 36 bp in the human genome. However, the presence of such motifs in the genome is not sufficient for AID binding both in vitro and in vivo.^12,13^

The DNA deamination activity of AID has been shown to be tightly coupled with transcription,^7^ which exposes enormous ssDNA substrates as potential AID-binding sites. To explain AID targeting specificity, several models have been proposed.^14^ First, early DNA targeting models propose that AID gains access to ssDNA either by directly associating with the RNA Pol II pausing/stalling cofactor Spt5^15^ or via the ssDNA-binding protein RPA (replication protein A).^16^ As ~60% of expressed genes in B cells show Spt5 occupancy,^15^ it is still unclear why only a very small subset of them are direct targets of AID. RPA is an ssDNA-binding protein and plays pivotal roles in DNA replication, recombination and repair.^17^ Theoretically, RPA will occupy the entire ssDNA region during transcription or replication, and thus how it specially recruits AID at some sites but not on other loci in the genome is unknown. Second, RNA exosome and 14-3-3 adaptor proteins have been shown to stimulate AID deamination activity at switch regions^18,19^; however, whether these two factors determine AID genome-wide targeting is still elusive. Third, two recent studies suggest that RNA or DNA at switch regions may form complex secondary structures, such as G-quadruplexes, to guide AID targeting^20,21^; but whether these structures are present at physiological conditions^22^ and how they might function to facilitate AID targeting are not known. Therefore, none of the existing models explains how AID is specifically loaded onto restricted ssDNA sites in the genome.

Besides *Ig* loci, AID also promiscuously mutates a large number of non-*Ig* targets,^23–25^ such as proto*-*oncogenes *BCL6*, *Myc*, *Pim1*, *Pax5*, and *PVT1*,^26–30^ leading to oncogenic mutations and chromosome translocations, which is thought to be a major cause of leukemia and lymphoma.^14,23,31^ It has been estimated that ~25% of expressed genes in germinal center B cells might be direct targets of AID.^24^ Such promiscuous activity of AID seems to be correlated with highly transcribed regions, such as super-enhancers and promoters, both of which are active in transcribing large amounts of non-coding RNAs, thereby exposing ssDNA substrates for AID targeting.^32–34^ As over 90% of mammalian genes show bi-directional transcription at promoter and enhancer regions,^35^ yet AID deaminates only a small fraction of these genes, it has been unclear whether those bi-directionally transcribed RNAs directly participate in AID recruitment.

Here we demonstrate the requirement for ROD1 to mediate both CSR and SHM in activated B cells. In both processes, ROD1 directly interacts with AID, which jointly bind bi-directionally transcribed RNAs to facilitate genome-wide AID targeting to *Ig* and non-*Ig* loci, suggesting that cooperative binding of the ROD1-AID complex on RNA provides the targeting specificity for AID. Moreover, we found that the C147X mutation observed in HIGM2 patients disrupts the interacting surface between AID and ROD1, leading to a failure in CSR. These findings thus unveil a completely unexpected disease mechanism, and demonstrate the functionality of bi-directionally transcribed RNAs in AID loading, which is fundamentally distinct from the elucidated roles of RPA, Spt5, RNA exosome, and 14-3-3 proteins in AID recruitment.

## Results

### Tethering AID to RNA induces active deamination in DNA

**Fig. 1:**
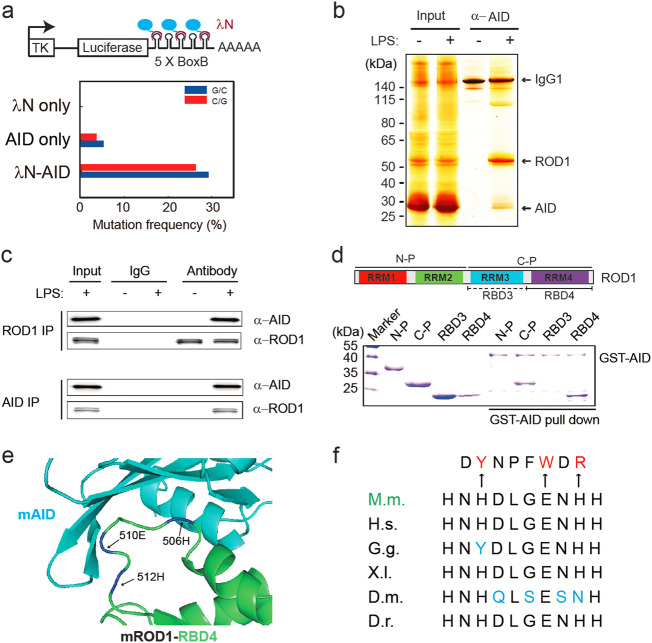
ROD1 physically interacts with AID via an ultraconserved loop region. **a** Diagram of the λN/BoxB tethering assay and the mutation frequency observed in HEK293 cells. The C/G mutations to all C/G bases in BoxB region were calculated from 20 sequenced clones. **b** Silver staining of AID immunoprecipitates from lysates of either LPS-activated or naive splenic B cells. **c** ROD1 and AID interact with each other in LPS-activated B cells. The reciprocal co-IP was probed with anti-AID and anti-ROD1 antibodies. **d** Direct interaction between AID and ROD1 truncated proteins by GST pull-down assay. RRM RNA recognition motif, N-P N-terminal protein, C-P C-terminal protein, RBD3 RNA-binding domain 3, RBD4 RNA-binding domain 4. **e** The 3D interacting surface of AID (cyan) and ROD1 (green) modeled by PRISM. The key interacting amino acids are labeled in blue and indicated by arrowheads. **f** The residue composition and conservation of the loop region in ROD1. Amino acids from 504 to 513 were aligned across the animal kingdom. The mutated amino acids at each position are listed and marked by arrowheads. D.r. zebrafish, D.m. fly, X.I. frog, G.g. chicken, H.s. human, M.m. mouse

With the guiding of sgRNA and the dsDNA unwinding activity of dCas9, AID can be directly tethered to dsDNA to induce site-specific mutations.^36^ This RNA-guided system prompted us to consider a possibility that a similar strategy might be naturally employed in activated B cells to impart AID specificity via newly transcribed RNAs, which would be in line with the observation that the GST-AID fusion protein is more efficiently cross-linked by UV to RNA than DNA.^8^ To test this idea, we performed a λN/BoxB tethering assay,^37^ in which multiple BoxB elements were inserted into RNA generated from a reporter and AID was fused to λN to recognize those BoxB elements, thereby forcing AID to newly synthesized RNA in HEK293 cells. Strikingly, compared to AID-only, we found that λN-AID, but not λN alone, caused ~30% C/G mutations in the BoxB region (Fig. 1a). To mimic the AID action in the context of chromatin, we further integrated the BoxB-containing reporter into the genome of the CH12F3 lymphocyte cell line (Supplementary information, Figure S1a). Again, we detected ~10% C/G mutations in response to λN-AID transduction, but not λN alone (Supplementary information, Figure S1b). Moreover, we observed a similar mutational spectrum in transfected HEK293 cells, indicating that G:C/A:T transitions and secondary mutations accumulated in vivo (Supplementary information, Figure S1c). These data suggest that RNA tethering is sufficient to guide AID to induce cytidine deamination in ssDNA.

### RNA-binding protein ROD1 physically interacts with AID

Since AID does not seem to have specificity in RNA binding in vitro,^6,8^ we speculate an uncharacterized co-factor(s) may exist and help define the AID targeting specificity in B cells. Given the potential involvement of RNA, we further speculate that such factor may correspond to an RNA-binding protein (RBP). Indeed, by performing an unbiased proteomic screening, we identified a unique candidate, ROD1 (*Regulator of Differentiation 1*),^38^ as an AID-associated factor in LPS-activated B cells, but not in naive B cells (Fig. 1b; Supplementary information, Table S1). As ROD1 was also highly expressed in B cells and mainly localized in the nucleus (Supplementary information, Figure S2a-c), we next performed coimmunoprecipitation (co-IP), which showed that endogenous ROD1 and AID were able to interact with each other in LPS-activated B cells (Fig. 1c). The interaction was independent of DNA or RNA since treatment of DNase I or RNase A had no influence on the interaction between Flag-tagged ROD1 and HA-tagged AID in transfected HEK293 cells (Supplementary information, Figure S3a). We further demonstrated the direct interaction between ROD1 and AID using bacterially expressed His-ROD1 and GST-AID in an in vitro pull-down assay (Supplementary information, Figure S3b).

To determine which domain in ROD1 was responsible for physical interaction with AID (Fig. 1d), we tested a series of truncation mutants of ROD1, revealing that the C-terminus of ROD1 containing RBD4 (RNA-binding domain 4) directly binds AID (Fig. 1d). Based on the available structural data for both AID and ROD1,^39,40^ we modeled their complex structure, suggesting that an ultraconserved loop in RBD4 of ROD1 is able to fit into a pocket structure in AID (Fig. 1e, f; Supplementary information, Figure S3c). Indeed, a loop-deleted form of ROD1 failed to interact with AID (L-D, Supplementary information, Figure S3d), and the residues H506, E510 and H512 were all found to be critical for ROD1 binding to AID (Supplementary information, Figure S3d).

Demonstrating that ROD1 and AID interact with each other in vitro and in vivo, we next tested whether ROD1 was able to regulate AID targeting. For this purpose, we performed chromatin immunoprecipitation and quantitative PCR (ChIP-qPCR) with a specific antibody against AID on 16 *Ig* and non-*Ig* targets, including well-characterized *IgH*, *miR-142*, *Pax5*, *Pim1*, *c-Myc*, *Cd79b*, and *Cd83* loci. For comparison, we chose 5 AID non-targets as negative controls. Upon stimulation of primary B cells by LPS, the AID occupancy on all 16 targets was significantly increased by at least two-fold compared to naive B cells, and by contrast, we only detected background signals on the 5 non-target loci (Supplementary information, Figure S4a, b). Moreover, enriched ChIP signals on AID targets were completely lost upon depletion of ROD1 with shRNA, which could be rescued by a shRNA-resistant form of ROD1 but not a loop deletion mutant (Supplementary information, Figure S4a), thus highlighting the functional importance of the ultraconserved loop region for AID interaction. Together, these data suggest that ROD1 directly interacts with AID, which might contribute to AID targeting in mammalian genomes.

### Defective CSR and SHM in *ROD1*^−/−^ mice

**Fig. 2:**
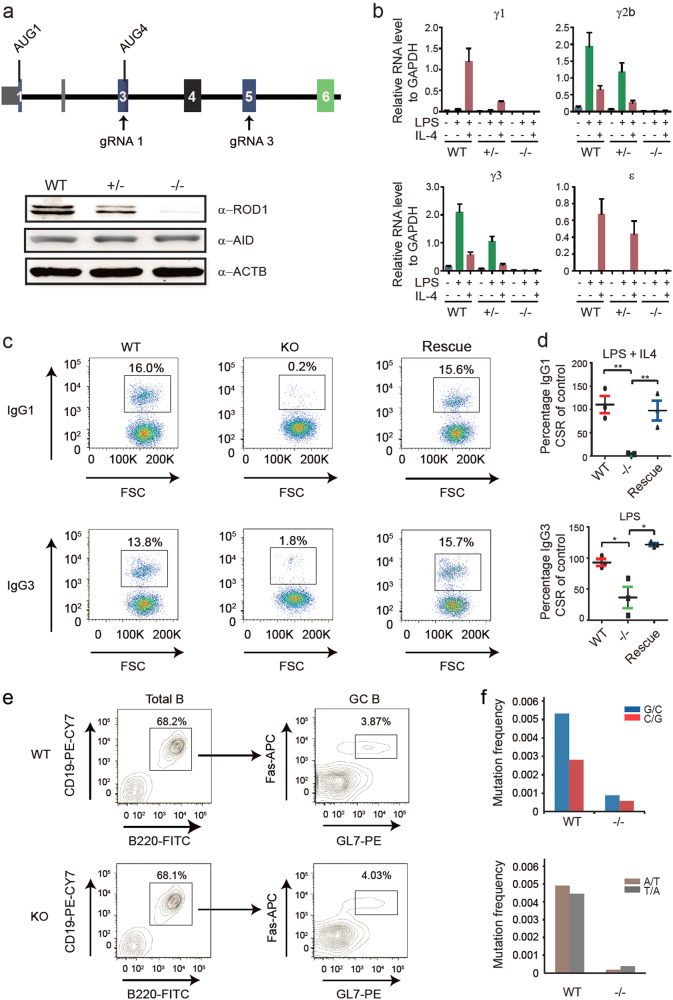
CSR and SHM are impaired in *ROD1*^*exon3*−/−^ mice. **a** The absence of ROD1 protein in *ROD1*^*exon3*−/−^ B cells. Diagram of gRNAs and the major AUG start codon in the *ROD1* loci (upper panel). ROD1 and AID expression were examined by Western blotting. β-actin served as the loading control. **b** RT-qPCR examination of post-switch transcripts containing Iμ and each of the C_H_ exons. The RNA levels were normalized to GAPDH (*n* = 3). **c** Flow cytometric analysis of IgG1 and IgG3 with ex vivo cultured splenic B cells activated either by LPS or LPS plus IL4. Splenic B cells were isolated from wild-type and *ROD1*^*exon3*−/−^ mice. Exogenous ROD1 was transduced into *ROD1*^*exon3*−/−^ B cells before stimulation to rescue CSR defects (*n* = 3). **d** Quantification of IgG1 and IgG3 CSR efficiency by normalizing to WT levels as shown in (**c**). *P < 0.05, **P < 0.01 (two-tailed Student’s *t* test). **e** Flow cytometric analysis of Peyer’s patch germinal center B cells in wild-type (WT) and ROD1 knockout (KO) mice. Percentage of GL-7^+^/Fas^+^ germinal center B cells amongst all B220^+^ cells is indicated. **f** SHM analysis of Peyer’s patch germinal center B cells at AID hypermutation substrates located at the JH4 intron. The mean values were determined by Sanger sequencing from 73 (WT) and 88 (−/−) sequenced clones

If ROD1 is responsible for AID targeting, we would expect to detect defective CSR and SHM in the absence of ROD1. To functionally test this possibility, we generated two strains of *ROD1*-deficient mice by disrupting either exon 3 or exon 5 with two different gRNAs (Fig. 2a). Western blotting confirmed the intended ablation of ROD1, which has no influence on AID expression levels (see Fig. 2a, bottom panel). Similar to *AID*-deficient mice, *ROD1*^−/−^ mice were viable and fertile, with normal spleen, lung, testis and thymus structures (Supplementary information, Figure S5a-c). Little difference was observed in hematopoietic cell populations from spleen between *ROD1*^+/+^, *ROD1*^+/^^−^ and *ROD1*^−/−^ mice (Supplementary information, Figure S5d). Strikingly, while the IgM level was similar, the levels of IgA, IgG2b, IgG3, IgE, and IgG1 were significantly decreased in *ROD1*^−/−^ mice compared to *ROD1*^+/+^ mice (Supplementary information, Figure S6a), which largely phenocopied the defects in *AID*^−/−^ mice.^2^ Of note, the reduced serum antibody levels were not due to defects in cell proliferation as CFSE labeling showed no detectable changes in highly purified naive splenic B cells from *ROD1*^+/+^ and *ROD1*^−/−^ mice (Supplementary information, Figure S6b-d).

We next evaluated the CSR efficiency in ex vivo cultured splenic B cells upon *ROD1* ablation. In response to LPS or LPS plus IL-4 stimulation, all post-switch transcripts were barely detectable in *ROD1*-deficient B cells (Fig. 2b), while germline transcripts did not exhibit any change (Supplementary information, Figure S6e), suggesting a B cell-specific CSR defect in the absence of T cells. Consistent with the reduced post-switch transcripts in *ROD1*^−/−^ mice, upon LPS or LPS plus IL-4 stimulation, we found that the abundance of IgG1, IgG3, IgG2b and IgE were also dramatically reduced in *ROD1*-deficient B cells (Fig. 2c, d; Supplementary information, Figure S6f, g). Importantly, such defects could be rescued by exogenously expressed ROD1 (Fig. 2c, d; Supplementary information, Figure S6f, g), thus excluding the possibility of off-target effects by CRISPR-Cas9 used to disrupt *ROD1*.

To examine SHM efficiency in the absence of ROD1, we isolated Peyer’s patches from the small intestine of *ROD1*^+/+^ and *ROD1*^−/−^ mice (see methods). The germinal centers in *ROD1*^−/−^ mice showed normal sizes and morphologies compared to those in *ROD1*^+/+^ mice by hematoxylin and eosin staining (data not shown). We also performed immunostaining with IgD and GL7 to discriminate follicular and germinal center B cells, respectively; and again, observed no obvious morphological changes in germinal centers (data not shown). Notably, though the percentages of GL7^+^/Fas^+^ germinal center B cells were roughly similar between *ROD1*^+/+^ and *ROD1*^−/−^ mice (Fig. 2e), the total mutation frequency at JH4 intron, a well-known AID substrate in germinal center B cells, was dramatically reduced upon *ROD1* ablation (Fig. 2f). Together, these data indicate that ROD1 is required for both CSR and SHM.

### B cell autonomous effect of ROD1 on CSR

As T cells also play important roles in B cell activation, we wished to further demonstrate the B cell autonomous effect of *ROD1* ablation on induced class switching by two bone marrow transplantation (BMT) strategies. In the first BMT strategy, bone marrow (BM) cells from CD45.1^+^ wild-type (WT) mice were mixed 1:1 with BM cells from CD45.2^+^ ROD1 KO mice followed by transplantation into CD45.1^+^/CD45.2^+^ recipients (Supplementary information, Figure S7a). Compared to CD45.1^+^ WT B cells, we detected similar percentages and populations of CD45.2^+^ B cells in both the BM and spleen in mixed chimeric mice (Supplementary information, Figure S7b-d and S8). These data suggest that ROD1 ablation did not impair B cell viability and differentiation. In the second BMT strategy, BM cells from age-matched muMT mice, which lack mature B cells but possess normal T cells,^41^ were mixed at a ratio of 4:1 with BM cells from either *ROD1*^+/+^ or *ROD1*^−/−^ mice and then transplanted into lethally irradiated recipients (Supplementary information, Figure S9a). Among the resultant muMT and *ROD1*^−/−^ chimeric mice, most, if not all, of T cells were normal, but ROD1 in B cells was specifically depleted. After 6 weeks of BMT, we found that, while ROD1 depletion had no influence on B cell differentiation (Supplementary information, Figure S9b-d and S10), the serum titers of antibodies were significantly reduced in both *ROD1*^*exon3*−/−^ and *ROD1*^*exon5*−/−^ mice compared to those in *ROD1*^+/+^ mice (Supplementary information, Figure S11). Together, these data clearly show a B cell autonomous effect of ROD1 on CSR.

### ROD1 directs AID targeting genome wide

**Fig. 3:**
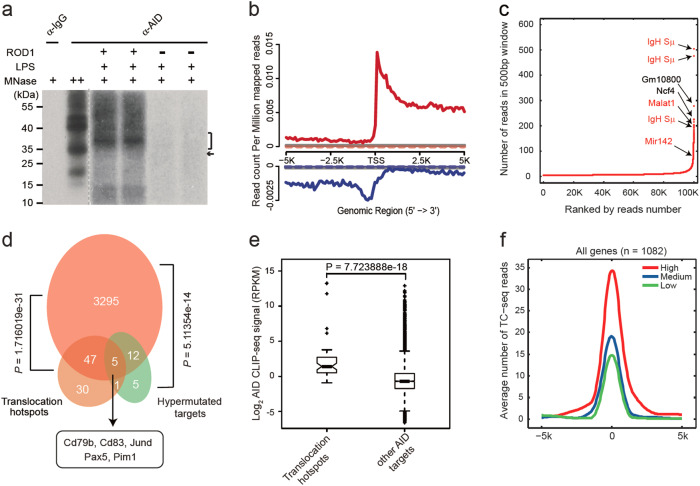
AID CLIP-seq in LPS-activated B cells. **a** Autoradiograph of ^32^P-labeled RNA cross-linked to AID. AID CLIP-seq was performed on LPS-activated B cells in the presence (+) or absence (−) of ROD1. RNA-protein complexes of 35 kDa under high micrococcal nuclease conditions corresponding to AID and short cross-linked RNAs (marked with an arrowhead). **b** AID read density from −5 kb to +5 kb of the TSS. The AID profile is shown as either solid or dashed lines based on the presence or absence of ROD1. The IgG profile is shown as gray line. The upper panel is the signal for the Watson strand (red), whereas the bottom panel is for the Crick strand (blue). **c** AID target ranking based on CLIP-seq binding strength. AID CLIP-seq signals are ranked by the number of unique reads in a 500-bp window. The top hits of several reported AID targets are marked by arrowheads and labeled in Red. **d** Venn diagram showing overlapped genes between AID CLIP-seq targets, chromosomal translocation hotspots and somatic hypermutation targets. *P* values were calculated by Fisher’s exact test. **e** Enrichment of AID CLIP-seq reads among translocation hotspots in LPS-activated B cells. The comparison is performed between 52 translocation hotspot genes and 3,307 other AID targets. *P* value was determined by Wilcoxon rank-sum test. **f** AID-binding profiles around the center of the translocation hotspots from −5 kb to +5 kb for all the TC-seq detected genes in B cells. TC-seq detected genes (*n* = 1082) were subgrouped into low, medium, or high based on the AID CLIP-seq signals

Since both ROD1 and AID are required for CSR and they form a complex in vivo, we next examined whether ROD1 regulates the genome-wide targeting of AID. To this end, we mapped AID in vivo targets by Crosslinking and Immunoprecipitation sequencing (CLIP-seq) in the presence or absence of ROD1 in LPS-activated B cells. Strikingly, the AID-RNA interaction was largely lost upon ROD1 depletion (Fig. 3a), which was not due to RNA degradation, as the nascent RNA production in both WT and *ROD1*-ablated B cells was similar (Supplementary information, Figure S12a). We proceeded to generate two highly reproducible AID CLIP-seq libraries (Supplementary information, Figure S12b, c). Interestingly, we found that AID generally preferred transcription start sites (TSSs) active in generating bi-directional transcripts and the AID CLIP-seq signals were lost in *ROD1*-ablated B cells, suggesting that ROD1 is specifically required for AID binding to RNA (Fig. 3b).

Unexpectedly, we identified 15,344 high-confidence AID peaks, 9442 of which were clustered in 3359 genes (Supplementary information, Table S2), which were more than expected.^14^ To examine the functional relevance of the CLIP-seq targets, we first ranked the uniquely mapped AID CLIP-seq reads in 500-bp windows genome-wide, and consistent with its primary loading at switch regions, the top two most enriched loci were *IgH Sμ* (Fig. 3c). Next, we explored whether previously reported AID targets could also be captured by CLIP-seq.^24,42^ Indeed, the newly identified target genes showed a significant overlap with recurrent AID-dependent translocations (*n* = 83) and hypermutations (*n* = 23) (*P* < 5.114 e-14, Fig. 3d; Supplementary information, Table S3). Moreover, genes containing translocation hotspots showed much stronger AID CLIP-seq signals compared to those without hotspots (Fig. 3e). These data strongly suggest that CLIP-seq mapped regions correspond to physiological substrates of AID.

AID-mediated chromosome translocation to *IgH* or *Myc* in B cells has been identified by translocation capture sequencing (TC-seq) or genome-wide translocation sequencing (HTGTS).^42,43^ Of note, the TC-seq or HTGTS targets were identified by I-SceI meganuclease-mediated cleavage at *c-myc* or *IgH* locus, and thus, most detected translocations were one-to-all events in the genome. This contrasts our all-to-all datasets, by which we identified in vivo AID targets by directly mapping its binding positions at all transcribed RNAs, including both translocation targets and potential mutation sites. To further compare these two datasets, we downloaded the TC-seq data and identified 1082 AID-mediated translocation events to *IgH*. We then divided TC-seq targets into low, medium and high subgroups based on AID CLIP-seq density, and interestingly, we found that if a genomic locus showed more RNA binding by AID, it tends to be more prone to translocate to other chromatin loci (Fig. 3f). Importantly, AID-binding events are preferably centered on translocation hotspots (Fig. 3f), suggesting that CLIP-seq mapped sites faithfully capture AID targets in vivo and the RNA-binding characteristics may be useful for inferring AID activities. Together, the above data strongly suggest that ROD1 confers genome-wide AID targeting in LPS-activated B cells.

### ROD1 and AID prefer bi-directionally transcribed regions

**Fig. 4:**
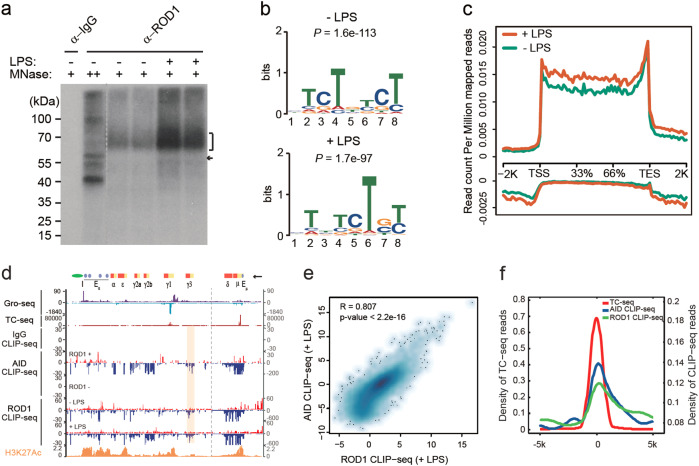
ROD1 and AID CLIP-seq signals are highly correlated. **a** Autoradiograph of ^32^P-labeled RNA cross-linked to ROD1. A single RNA-protein band of 57 kDa under high micrococcal nuclease conditions corresponding to ROD1 and short cross-linked RNAs (marked with an arrowhead). **b** Consensus motifs identified by CLIP-seq in naive or LPS-activated B cells. **c** ROD1 CLIP-seq read density around the TSS, gene body, 3′end and intergenic regions. The upper and bottom panels show the signals for the Watson and Crick strands, respectively. **d** Snapshot of Gro-seq, TC-seq, and CLIP-seq profiles at the *IgH* locus in B cells stimulated with LPS or not. Above, the *IgH* locus is annotated with the S-regions (yellow) and C-regions (red boxes) as well as the 5′ μ-chain enhancer (Eμ), 3′ α-chain enhancer (Eα), and insulator (I). The arrowhead indicates the transcription direction. The gray dash line separates the *IgH* locus into two parts with different y-axis scales. The AID and ROD1 bindings at γ3 are labeled by the pink box. **e** The correlation analysis of AID and ROD1 CLIP-seq in LPS-activated B cells. Scatter plots represent log_2_-normalized RPKM values of Refseq genes between the two datasets. The *P* value and *R* (Pearson’s correlation efficient) are indicated. **f** The CLIP-seq profile of AID and ROD1 around the center of the translocation positions detected by TC-seq

To dissect how ROD1 mediates AID targeting, we also mapped ROD1-binding sites in naive and LPS-activated B cells (Fig. 4a). After pooling two highly reproducible replicates, we obtained 60 and 65 M mapped reads for ROD1 from LPS-treated and mock-treated cells, respectively (Supplementary information, Figure S13a). MEME motif analysis revealed a consensus sequence of UCUCUCU before and after LPS stimulation (Fig. 4b). Interestingly, the ROD1-binding profiles in the mouse genome were similar before and after LPS stimulation (Fig. 4c; Supplementary information, Figure S13b, c), indicating that ROD1 resides in AID targeting sites prior to B cell activation. Importantly, we found a strong positive correlation between ROD1 and AID CLIP-seq signals at individual sites (Fig. 4d; Supplementary information, Figure S13c) as well as on the genome-wide scale (*R* = 0.807, *P* < 2.2e-16, Fig. 4e; Supplementary information, Table S2 and Table S4). It has been established earlier that AID is preferentially recruited to different switch regions upon different stimulations.^19^ Indeed, we found that ROD1 preferred to bind the primary transcripts of S-γ3 but not S-γ1 upon LPS stimulation (Fig. 4d, pink boxed region). In contrast, ROD1 and AID predominantly bound S-γ1 rather than S-γ3 upon stimulation of LPS plus IL-4 (Supplementary information, Figure S14). Like AID, the ROD1 CLIP-seq profile also showed a significant positive correlation with TC-seq and Gro-seq signals (Fig. 4f; Supplementary information, Figure S15). Together, these data suggest that ROD1 may specify AID targets by binding to nascent RNA.

**Fig. 5:**
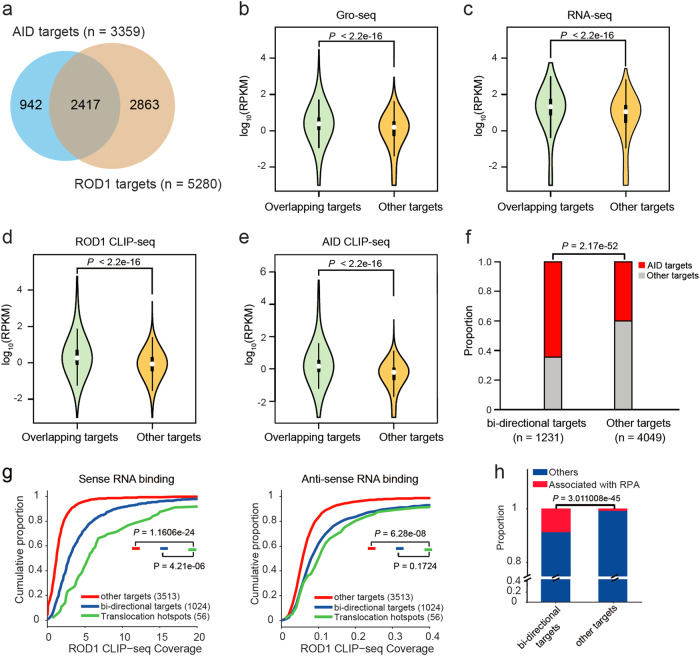
The overlapping targets of AID and ROD1 tend to be bi-directionally transcribed. **a** Venn diagram showing the overlapping targets between AID and ROD1 by CLIP-seq. **b**, **c** Violin plots showing the distributions of RNA levels measured by Gro-seq or RNA-seq for both overlapping and non-overlapping target sets (other targets). **d**, **e** Violin plots showing the distributions of ROD1 or AID CLIP-seq read density for both overlapping and non-overlapping target sets. The *P* values were calculated by unpaired Student’s *t* test (**b**–**e**). **f** Bi-directionally transcribed ROD1 targets tend to be co-occupied by AID. ROD1 targets were subgrouped into bi-directional targets and other targets. The overlapping AID targets (*n* = 2417, red) show a significant enrichment for the bi-directionally transcribed group. **g** CDF plot of ROD1 CLIP-seq coverage on the sense (left) or antisense (right) strand of the three grouped genes. Notably, some genes are removed from the bi-directional target group because they are close to each other in 2 Kb windows, making it difficult to assign RNA signals to a single gene. The Kolmogorov–Smirnov test was applied. **h** ROD1 bi-directional targets share significant overlap with AID targets as revealed by RPA ChIP-seq. Fisher’s exact test was applied

CLIP-seq mapping revealed ~5200 highly reproducible in vivo targets of ROD1, 2417 (~46%) of which directly overlapped with AID CLIP-seq targets (Fig. 5a); thus we next explored the signatures of their overlapped targets compared to non-overlapped (others, *n* = 2863) targets. We found that overlapped genes tend to be more actively transcribed (Fig. 5b, c), and have stronger CLIP-seq signals (Fig. 5d, e). Furthermore, ~23.6% of the ROD1 targets overlapped with bi-directional transcripts (Fig. 5f), including those associated with super-enhancers and promoters.^32–34^ By comparing the distribution of AID CLIP-seq targets (overlapped with ROD1, *n* = 2417) in ROD1 bi-directional target group vs. non bi-directional target group (others), we found that AID targets show significant overlap with bi-directional targets of ROD1 (*P* = 2.17e-52, Fig. 5f).

Given that ROD1 binding prefers promoters and enhancers that show strong bi-directional transcription, we further divided ROD1 targets into two groups, one with bi-directional ROD1 binding and the other without such feature. By analyzing ROD1 binding to sense or antisense RNAs, we found that ROD1 bi-directional targets with preferred antisense RNA binding were more likely associated with AID recruitment, and the curve was similar to that generated with 56 well-known AID translocation hotspot genes (*P* = 0.1724, Fig. 5g). We next explored whether the bi-directional ROD1-binding feature could be used to predict AID activity. For this purpose, we chose another group of AID targets indirectly deduced by RPA-seq (*n* = 203, see Supplementary information, Table S3) as input,^34^ and interestingly, we found that more RPA-seq targets overlap with bi-directional ROD1-binding targets (*P* = 3.011008e-45, Fig. 5h; Supplementary information, Table S3), indicating that the bi-directional binding feature of ROD1 can be used to predict AID activities genome wide. Together, these data suggest that ROD1 binding on bi-directionally transcribed regions lead to more active recruitment of AID.

### ROD1 specifies AID-loading sites via RNAs and CU-rich motifs

The binding of ROD1 to bi-directional RNA transcripts prompted us to consider the possibility that these nascent transcripts might function as a stepping stone for ROD1 to recruit AID. To test this idea, we first investigated whether ROD1’s access to AID targets also depends on active transcription. For this purpose, we used two drugs (D-rybofuranosylbenzimidazole (DRB) or flavopiridol) to briefly inhibit RNA Pol II elongation for 4 h. Upon such a short time treatment, the steady-state levels of ROD1 and AID were unchanged (Supplementary information, Figure S16a); however, the occupancy of ROD1 on 10 well-characterized AID targets was sharply reduced, indicating that ROD1 may recruit AID in a transcription-dependent manner (Supplementary information, Figure S16b).

**Fig. 6:**
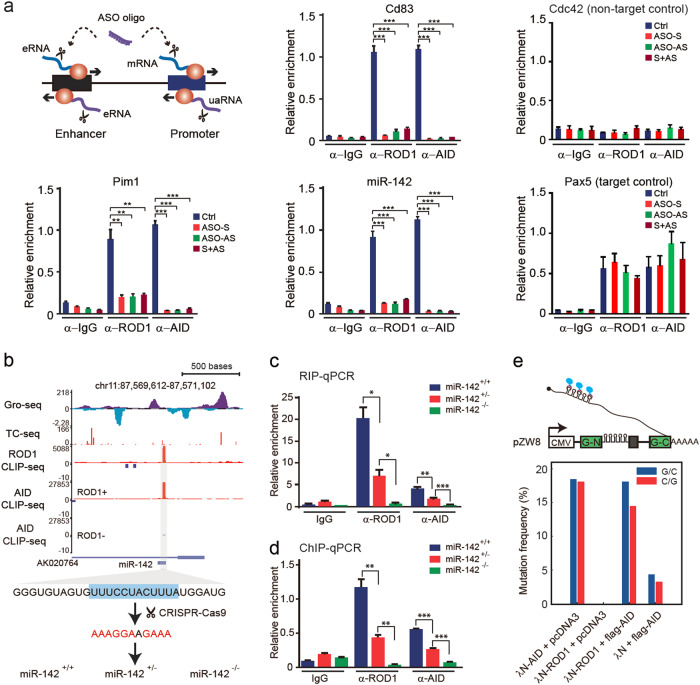
ROD1 cotranscriptionally recruits AID via bi-directional RNA. **a** ChIP-qPCR analysis of ROD1 and AID occupancy in AID target and non-target regions upon depletion of bi-directional RNA. Gene-specific ASOs to *Cd83*, *Pim1*, and *miR-142* were individually transfected into LPS-activated B cells for 48 h. *Cdc42* served as an AID non-target control, whereas *Pax5* as AID target control, both of the samples were treated by CD83-ASO oligos. The relative enrichment was calculated by normalizing all of the data against input DNA. Data are presented as the mean ± SD (*n* = 3). The cartoon depicts bi-directional transcription at promoters and enhancers in mammalian cells. eRNA enhancer RNA, uaRNA promoter upstream antisense RNA. **b** Snapshot of the Gro-seq, TC-seq, and CLIP-seq profiles in the vicinity of the *miR-142* locus. The ROD1-binding sequence highlighted in cyan was mutated into AG-rich sequences by CRISPR-Cas9 in CH12F3 cells. **c**, **d** RIP-qPCR or ChIP-qPCR analysis of ROD1 and AID occupancy at the *miR-142* locus in different genotypes. The data are presented as the mean ± SD (*n* = 3). **e** Diagram of the λN/BoxB intronic tethering assay and the mutation frequency observed in HEK293 cells. The C/G mutation frequency was determined by Sanger sequencing from 20 sequenced clones. *P < 0.05, **P < 0.01 and ***P < 0.001 by two-tailed Student’s *t* test

We next tested whether transcription-generated RNAs are critical for ROD1 to recruit AID. To the end, we exploited ASO oligonucleotides to knockdown either sense or antisense nascent RNAs at promoter or enhancer regions (see illustration in Fig. 6a), and then examined ROD1 and AID occupancy on the *miR-142*, *Cd83*, and *Pim1* loci by ChIP-qPCR. In contrast to the non-target control *Cdc42* and the target control *Pax5*, the levels of ROD1 and AID bound to these chromatin regions were significantly decreased upon depletion of sense or antisense RNA (Fig. 6a; Supplementary information, Figure S16c). These data suggest that bi-directionally transcribed RNAs are critical for the binding of ROD1 and AID to chromatin.

To further investigate whether AID recruitment depends on ROD1-binding motif (CU-rich), we chose one of the well-known AID targets, *miR-142*, as a model because the strong and sharp binding signals for both ROD1 and AID (Fig. 6b). Using CRISPR-Cas9, we successfully mutated the ROD1 CU-rich binding motif into a GA-rich sequence in CH12F3 cells without changing primary *miR-142* expression (Fig. 6b; Supplementary information, Figure S16d). RIP-qPCR analysis revealed that ROD1 binding to RNA was dramatically reduced in both heterozygous and homozygous cells (Fig. 6c). Correspondingly, the levels of AID binding to chromatin became almost undetectable in homozygous cells (Fig. 6d), suggesting a sequence-dependent recruitment of ROD1, and subsequently, AID. These data also support the idea that ROD1 may bind first to nascent RNAs and then recruit AID.

As ROD1-mediated AID targeting relies on active transcription and specific RNA motif in nascent RNA, we hypothesize that ROD1 may load AID onto chromatin in a co-transcriptional manner. To test this idea, we first inserted five copies of BoxB sequence into an intronic region of a splicing reporter, and then performed a λN-BoxB tethering assay to mimic the cotranscriptional targeting process because splicing is known to occur cotranscriptionally.^44,45^ As expected, we found that tethering λN-ROD1 to the intronic region of the reporter did not cause any mutations in BoxB regions (Fig. 6e, column 2), because AID is not expressed in HEK293 cells (Supplementary information, Figure S17). Remarkably, however, tethering ROD1 in the presence of AID achieved roughly similar C/G mutation frequencies as tethering λN-AID (Fig. 6e, comparing column 1 and column 3), indicating that ROD1-mediated AID targeting is a highly potent process. Moreover, the C/G mutation frequency (~20%) in these two groups is ~five-fold higher than the background mutation rate caused by ectopic expression of Flag-tagged AID (Fig. 6e). Thus we conclude that ROD1 cotranscriptionally loads AID via nascent RNAs.

### Disease-associated AID mutations disrupt the interaction with ROD1

**Fig. 7:**
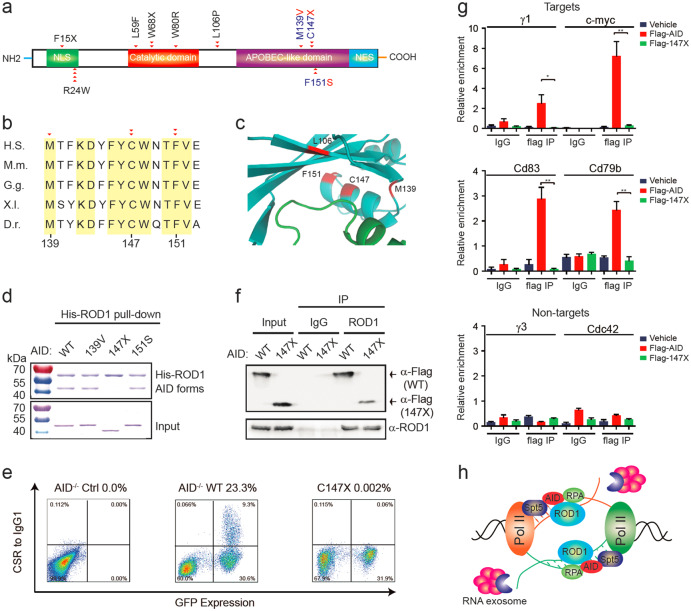
HIGM2 mutations in AID disrupt its interactions with ROD1. **a** Diagram of the domain structure of AID and HIGM2-related mutations. Naturally occurring mutations in patients (red triangle) from a previous report^46^ are shown at the exact residue positions. **b** The mutation residues are highly conserved. Amino acids from 139 to 153 were aligned. D.r. Zebrafish, X.l. frog, G.g. chicken, H.s. human, M.m. mouse. **c** The interacting surface between human AID and ROD1 modeled by PRISM. The mutations are marked in red. **d** His-ROD1 pulled-down GST-AID and some variant proteins, but not 147X variant. **e** Rescue of IgG1 CSR in *AID*^−/−^ splenic B cells by transducing Flag-tagged AID or AID^147X^ mutant. Percentage of IgG1 CSR is the ratio between IgG1^+^/GFP^+^ cells and total GFP^+^ cells. **f** Anti-ROD1 immunoprecipitates from retrovirally transduced *AID*^−/−^ B cells with either Flag-tagged AID or AID^147X^ mutant. **g** ChIP-qPCR analysis of AID and AID^147X^ mutant occupancy at S-γ1 and non-*Ig* targets in reconstituted *AID*^−/−^ B cells as shown in **f**. S-γ3 and *Cdc42* served as AID non-target controls. *P < 0.05 and **P < 0.01 by two-tailed Student’s *t* test. The data are presented as the mean ± SD (*n* = 3). **h** A model of bi-directionally trapped ROD1 recruiting AID. In naive B cells, ROD1 resides on bi-directionally transcribed RNA and waits for the expression of AID. Once AID is dramatically induced upon antigen stimulation, it will be guided to the defined sites to initiate CSR, SHM and translocations

Many mutations in the C-terminus of AID have been linked to an autosomal recessive immune disorder called HIGM2, which has been characterized by the absence of CSR and SHM in germinal center B cells, and correspondingly, patient sera usually lack IgA, IgE and IgG but have unchanged or higher levels of IgM.^46^ These mutations in AID have been classified into three groups: group 1 mutations are located in the nuclear localization signal (NLS); those in group 2 are clustered in the cytidine deaminase catalytic motif (residues 56–94); and those in group 3 are mapped close to the C-terminus (Fig. 7a). The first two group mutations disrupt AID nuclear localization and catalytic activities, respectively; however, how mutations in the third group cause HIGM2 has remained unclear.

Structural analysis revealed an N-terminal β-sheet (residues 40–43) and a C-terminal α-helix (residues 140–151) in AID, together forming a pocket structure (Fig. 1e; Supplementary information, Figure S3c). The α-helix and its vicinity are highly conserved from residues 139 to 151, which are frequently mutated in HIGM2 patients (Fig. 7a, b). Consistent with a recent report,^21^ we also found that the AID mutations in residues 140–151 still possess deamination activity (data not shown). These observations indicate that M139V, C147X and F151S may cause HIGM2 through other mechanisms (Fig. 7a). Considering their close proximity to the interacting surface with ROD1, we hypothesized that these mutations might impair the interaction between AID and ROD1 (Fig. 7c). To pursue this possibility, we used bacterially expressed His-ROD1 to pull down different AID mutant proteins, including AID M139V, AID C147X and AID F151S (Fig. 7d). Strikingly, we found that C147X mutation on AID completely disrupted its association with ROD1 (Fig. 7d). As X is a nonsense mutation, it is unclear whether the codon 147 or the amino acid sequence after amino acid 147 is important for contacting with ROD1. We thus changed C147 into other missense mutations, such as W, R and D, and found that all single and triple mutation variants failed to interact with ROD1 (Supplementary information, Figure S18a). These data suggest that C147 is the primary residue in AID responsible for specific interaction with ROD1.

We next determined to examine whether the mutation at residue 147 causes HIGM2. For this purpose, we tested the effect of C147X mutation on CSR in CH12F3 cells,^47^ which showed a highly efficient switch of the isotypes from IgM to IgA upon combined stimulation with CD40L, IL-4 and TGFβ (CIT). We successfully knocked in this mutation with CRISPR/Cas9 in these cells, and demonstrated that the mutation recapitulated the HIGM2 characteristics, such as unchanged IgM level, but nearly undetectable IgA upon CIT stimulation for 3 days (Supplementary information, Figure S18b). This suggests that C147X is a causal mutation for HIGM2.

To further explore the pathological mechanism underlying C147X mutation, we examined the interaction between ROD1 and AID C147X mutant in vivo. Because the anti-AID antibody we used was designed against the C-terminal region and efficient antibody against N-terminus is not available, we decided to use Flag-tagged WT or mutant proteins to reconstitute *AID*^−/−^ B cells. To this end, splenic B cells were isolated from *AID*^−/−^ mice, and infected with retroviral vectors expressing either AID-WT or the C147X mutant. Moreover, the retroviral vector also contains an IRES-GFP unit for tracing successfully transduced B cells. In contrast to *AID*^−/−^ B cells, which did not undergo CSR to IgG1 upon anti-CD40 plus IL-4 stimulation, the retroviral transduced AID-WT can efficiently induce CSR to IgG1 with an efficiency of 23.3%, indicating that such surrogated model could indeed restore AID activity in vivo (Fig. 7e). Under the same conditions and with the expression of roughly equal levels of AID-WT and the C147X mutant (Supplementary information, Figure S18c), we found that Flag-tagged C147X mutant failed to restore IgG1 CSR in *AID*-ablated B cells (Fig. 7e), thus further proving the causal effect of C147X in HIGM2. To examine the interactions between ROD1 and AID-WT or C147X mutant proteins, we collected the retroviral transduced B cells and performed co-IP with specific antibody against ROD1. Consistent with the His-ROD1 pull-down assay (Fig. 7d), we found that endogenous ROD1 barely interacted with the C147X mutant protein (Fig. 7f). These results demonstrated that the C147X mutation disrupts the interaction of AID with ROD1 both in vitro and in vivo.

As loading of AID onto IgH loci is essential for initiating the CSR process, we next examined whether the C147X mutant possesses the ability to find its *Ig* and non-*Ig* targets. To this end, we performed ChIP-qPCR analysis with anti-Flag antibody in AID-WT- or C147X mutant-reconstituted B cells. Upon stimulation of splenic B cells by anti-CD40 plus IL-4 to induce CSR to IgG1, we examined AID occupancy on S-γ1 and three non-*Ig* targets, including *c-myc*, *cd83*, and *cd79b*, with S-γ3 and *cdc42* as negative controls. In *AID*-deficient B cells, we only detected background ChIP signals in response to anti-CD40 plus IL-4, and upon reconstitution with Flag-tagged AID-WT, we observed strong signals of AID binding at both *Ig* and non-*Ig* loci, but the occupancy was completely lost if *AID*^−/−^ B cells were transduced with Flag-tagged C147X mutant (Fig. 7g), suggesting that the failure of this AID mutant in binding its targets in vivo is most likely due to severely reduced binding ability to ROD1. Together, these results indicate that the C147X mutation impairs CSR in HIGM2 patients due to the disruption of AID’s association with ROD1.

## Discussion

In summary, our data strongly suggest that bi-directionally transcribed RNAs from the proximal regions of enhancers and promoters function as a scaffold to trap ROD1, which altogether establish many “loading docks” in the mouse genome to guide AID targeting (Fig. 7h). This mechanism appears to be conserved in human Ramos B cells, as AID also failed to bind its target loci upon ROD1 depletion (Supplementary information, Figure S19). Moreover, our findings may provide a potential therapeutic target for HIGM2 and B cell lymphoma since the mutations that disrupt the “guiding system” cause an HIGM2-like phenotype (Fig. 7). In contrast to Spt5-, 14-3-3-, RNA exosome- and RPA-mediated DNA loading models, our RNA-mediated recruitment model elucidates the AID targeting specificity at three levels. First, bi-directional RNAs provide the first selection criterion. Second, ROD1 is highly selective in binding defined RNA sequences, further conferring the specificity for AID. Third, the conserved interacting surfaces between ROD1 and AID maintained the maximum extent of selectivity during evolution.

It is worth noting that ROD1 belongs to the PTB family of RNA-binding proteins, with the other two key members being PTB (encoded by the *PTBP1* gene) and nPTB (encoded by the *PTBP2* gene). Although PTB, nPTB and ROD1 share ~74% similarity in protein sequence, they are expressed in a tissue-specific manner. For example, PTB is ubiquitously expressed in most tissues except neurons,^48^ while its neuronal paralog nPTB is exclusively detected in differentiating neurons.^49,50^ We now discovered that the third paralog of the PTB family, ROD1, which was initially found to be predominantly expressed in hematopoietic cells,^38^ is highly restricted to B cells where it plays pivotal role in regulating antibody diversification. Interestingly, all PTB family members prefer binding to CU-rich sequence in RNA, but the temporal and spatial expression patterns determined their target repertories and regulatory functions in different mammalian cells. Thus, temporal and spatial expression of different RNA-binding proteins may further strengthens the specificity of diverse biological processes, including CSR and SHM processes.

Importantly, we have identified an ultraconserved loop region in ROD1 responsible for its direct interaction with AID. Though PTB contains the same loop sequence, we found that the expression of ROD1 is about five-fold higher than that of PTB in both naive and activated B cells (data not shown), indicating that ROD1 may be the primary partner for AID in B cells. Notably, as the neuronal paralog of ROD1,^50^ nPTB (also known as *PTBP2*) has been reported to interact with AID in CH12F3 cells.^51^ However, we failed to detect the expression of nPTB in B cells (data not shown), in line with the same observation from a recent work.^52^ Moreover, we found that nPTB contains a different loop sequence (HNYNLGENHH) from that in ROD1 (HNHDLGENHH), and the H506 in ROD1 is essential for its association with AID (Supplementary information, Figure S3d). Thus, theoretically, if nPTB can be expressed in B cells under certain circumstances, it should not possess the ability to directly interact with AID to initiate CSR. In addition, ROD1 has been shown to regulate alternative splicing and modulate mRNA stability.^53^ Future efforts are needed to determine how ROD1 confers its specificity to diverse tasks in B cells.

RNA-binding proteins regulate mRNA fate through a 4–8 nt regulatory RNA code.^54^ Their high selectivity for the RNA code provides an ideal strategy to determine the specificity in different functional contexts.^44^ As different RBPs tend to associate with one another to form various macromolecular complexes, each RBP in the complex may uniquely contribute to RNA code recognition, thus generating a combinational RNA code to institute the complex specificity observed in the cell.^55^ The AID “mutator complex” may contain other proteins that indirectly interact with AID,^56,57^ including several hnRNP proteins that work together with ROD1 to restrict AID targeting.

Recent genomic studies indicate that both promoters and enhancers are divergently transcribed, generating large amounts of antisense non-coding RNAs, such as eRNAs (enhancer RNAs) and uaRNA/xTSS-RNAs, which tend to be degraded by RNA exosome.^32,35^ Though RNA exosome has been shown to stimulate AID activity on both DNA strands,^18^ how it selectively loads AID onto a small subset of ssDNAs remains unclear. Our results also suggest that promoter- and enhancer-associated RNAs, are not by-products of transcription, but rather function as an adaptor to load diverse enzymes onto genomic DNA to modulate gene expression. This regulatory paradigm may thus be generally applicable to diverse biological processes.

## Materials and methods

### λN-BoxB tethering assay

For tethering assay, λN-Flag tag was synthesized and fused with AID or different ROD1 variants by PCR amplification with KOD hot-start DNA polymerase (Novagen, Catalog # 71086–5). λN, λN-Flag-AID and λN-Flag-ROD1 were inserted into pcDNA 3.0 at BamH1 site. To generate cotranscriptional reporter, 5× BoxB was amplified and inserted into pZW8 splicing reporter between BamH1 and Hind III. For transient tethering assay, λN variants and related 5× BoxB reporters were co-transfected into HEK293 cells with Lipofectamine 2000 (Invitrogen, Catalog # 11668–019). After 72 h, the genomic DNA was extracted according to manufacturer’s instruction (Selleck, Catalog # B40013). 5× BoxB was amplified and sequenced by Beijing Genomics Institutes (BGI). To generate CH12F3 stable cell lines, 5× BoxB reporter and λN or λN-Flag-AID were subcloned into pLenti CMV GFP puro (addgene, plasmid # 17448) or pLenti CMV GFP hygro (addgene, plasmid # 17446), respectively. These two plasmids were then packaged into lentiviral particles to infect CH12F3 cells. After these cells were selected with either puromycin (2 μg/ml, Clontech, Catalog # 631305) or hygromycin B (100 μg/ml, Invitrogen, Catalog # 10687010) and passaged for additional two times, the genomic DNA was extracted and 5× BoxB region was sequenced to examine mutation spectrum.

### Co-immunoprecipitation and mass spectrometry

Splenic B cells were isolated from 8-week-old mice by negative selection with EasyStep^TM^ mouse B cell isolation kit (Stem cell, Catalog # 19854). For immunoprecipitations, splenic B cells were lysed with NP40 lysis buffer (10 mM Tris-Cl, pH 7.4, 100 mM NaCl, 2.5 mM MgCl_2_, 0.5% NP 40) supplemented with 1 mM PMSF and 1× proteinase inhibitor cocktail (Sigma, Catalog # P8340) on ice for 15 min, and then briefly sonicated three times with BRANSON SLPe (output setting 4, 10 s per cycle). After spinning the lysed cells at 13,000 rpm for 20 min at 4 °C, the supernatant was transferred to a new eppendorf tube and the protein concentration was quantified using the BCA protein assay kit (Pierce, Catalog # 23227). Five hundred micrograms of proteins were diluted with NP40 lysis buffer to 1 μg/μl and pre-cleared with 10 μl of protein A/G magnetic beads (Pierce, Catalog # 88803) for 1 h at 4 °C; then the lysate was incubated with 10 μg of either anti-ROD1 or anti-AID antibody and rotated overnight at 4 °C. The samples were then incubated with 50 μl of protein A/G magnetic beads for 4 h at 4 °C. After thoroughly washing with NP40 lysis buffer for five times, AID or ROD1 enriched proteins were eluted with 1× LDS sample buffer for 10 min at 70 °C by vortexing at 1000 rpm with a Thermo Mixer C. The eluted proteins were then analyzed by Western blotting. For mass spectrometry analysis, 0.1 μg/μl of RNase A was further used to treat AID immunoprecipitates to remove RNA-mediated interaction partners. The enriched bands were cut and digested with trypsin. The digested peptides were identified by nano-ultra-performance liquid chromatography electrospray ionization tandem mass spectrometry. Data from liquid chromatography tandem mass spectrometry were processed using ProteinLynx Global Serverversion 2.4 (PLGS 2.4). The candidate peaks were used for searching with the Mascot search engine in the NCBI protein database.

### Knockout mice and cell line generation

*ROD1*^*exon3*−/−^ and *ROD1*^*exon5*−/−^ mice were generated by co-injection of Cas9 mRNA and gRNA into zygotes as previously described.^58,59^ All the surgical procedures were approved and performed under the guidelines of the IBP Animal Care and Use Committee. To generate AID^147X^ in CH12F3 cells, PX458-sgRNAs and donor plasmids were co-transfected with a Lonza electroporator (4D-Nucleofector system). After 48 h, GFP-positive cells were sorted into 384-well plates with one cell per well by FACS Jazz (BD) and AID^147X^ knock-in cells were confirmed by Sanger sequencing.

### Isotyping and enzyme linked immunosorbent assay (ELISA)

Mouse serum was collected from the orbital venous plexus, and the antibody in the serum was then measured following the manual of the mouse immunoglobulin isotyping kit (BD Biosciences, Catalog # 550026). The heavy and light chain isotypes of a mouse monoclonal antibody were analyzed by Fortessa. The immunoglobulin concentrations in naive mice were measured by mouse Ig ELISA quantitation sets in 96-well plates (Corning, Catalog # 3590) according to manufacturer’s protocol (Bethyl Laboratories, Catalog # E90-103, E90-115, E90-109, E90-111, E90-101, E90-105). Goat anti-mouse coating antibodies were 1:400 diluted and used for coating a 96-well plate at RT for 1 h. After blocking with 1% BSA in PBS for 30 min, mouse reference serum and samples were diluted and incubated in the assigned wells at RT for 1 h. The HRP-conjugated antibody was then applied and further incubated at RT for 1 h, after which, TMB substrate solution (BioLegend, Catalog # 421101) was added to each well and incubated at RT for 15 min, and then stopped by ELISA stopping solution (0.18 M H_2_SO_4_). The absorbance was measured on a micro plate reader at 450 nm (Tecan).

### Cell culture, CFSE labeling, and transfection

HEK293 cells were cultured in Dulbecco’s modified Eagle’s medium (DMEM, Gibco, Catalog # 11995065) supplemented with 10% fetal bovine serum (FBS, Gibco, Catalog # 16000044) and 100 U of Penicillin-Streptomycin in 5% CO_2_ at 37 °C. Transfections were performed with Lipofectamine 2000 (Invitrogen, Catalog # 11668–019) according to the manufacturer’s instructions. Splenic B cells were isolated from *ROD1* wild-type and *ROD1*^−/−^ mice following the manufacturer’s protocol for EasyStep^TM^ mouse B cell isolation kit. Primary B cells and CH12F3 cells were cultured in RPMI 1640 medium supplemented with 10% FBS and 100 U of Penicillin-Streptomycin. Primary B cells were activated by LPS (5 μg/ml, Sigma, Catalog # L3024) and/or IL-4 (20 ng/ml, Peprotech, Catalog # 214-4), whereas CH12F3 cells were stimulated by the combination of CD40L (0.5 μg/ml, Peprotech, Catalog # 100-21C), IL-4 (20 ng/ml) and TGF-β (20 ng/ml, Peprotech, Catalog # 315-15) to induce the class switch recombination from IgM to IgA. After 72 h stimulation, cells were analyzed by FACS sorting with biotin-conjugated anti-mouse IgA (Biolegend, Catalog # 407003) and fluorescein isothiocyanate (FITC)-conjugated anti-mouse IgM antibodies (Biolegend, Catalog # 406505). To CFSE labeling, 10^6^ of B cells were collected for each genotype and washed twice with PBS; the cell pellets were resuspended in 1 ml of PBS containing 5 μM CFSE (eBioscience, Catalog # 65-0850-85) and further incubated at RT for 2 min. The labeling was stopped by adding 6 ml of fetal bovine serum. After PBS washing, the naive (day 0) or LPS-stimulated (day 3) splenic B cells were quantified by flow cytometric analysis. ASO oligos were designed and synthesized by GenePharma. To deplete bi-directional nascent RNA, 300 pmol of sense or antisense oligo mixture were transfected into LPS-activated B cells with Lipofectamine RNAiMAX reagent (Life Technology, Catalog # 13-778-150).

### Class switch recombination

Splenic B cells from sex-matched 8-week-old mice were prepared by negative selection. The purities of the final isolated fractions typically range from 95 to 98% by analyzing B220 positive cell populations. The splenic B cells from *ROD1* WT or KO mice were ex vivo cultured for 16 h and then stimulated with LPS (5 μg/ml) or LPS plus IL-4 (20 ng/ml) for additional 72 h. To examine the levels of germline and post-switch transcripts, total RNA was extracted by TRIzol (Invitrogen, Catalog # 15596026) and reverse transcribed with M-MLV reverse transcriptase (Promega, Catalog # M1701). For class switch recombination, different post-switch transcripts were examined with previously published primer pairs.^2^ To rescue *ROD1* ablation induced CSR defects, splenic B cells from *ROD1* KO mice were infected by an exogenous *ROD1* expression unit for 24 h. LPS and/or IL-4 were then added to the medium of cultured B cells to induce class switch recombination. For IgE staining, mouse Fc receptors were first blocked with 1 µg BD Fc Block/10^6^ cells in 100 µl of staining buffer for 15 min at 4 °C (BD Fc Block ^TM^, catalog # 553142). The intracellular staining was then performed according to the manufacturer’s protocol (BD Cytofix/Cytoperm^TM^ fixation/permeabilization kit, Catalog # 554714). The following antibodies were used for flow cytometry analysis: anti-B220 (Biolegend, Catalog # 103232), anti-IgG1 (Biolegend, Catalog # 406605), anti-IgG3 (Biolegend, Catalog # 406803), anti-IgG2b (Biolegend, Catalog # 406707), and anti-IgE (Biolegend, Catalog # 406906). All the analyses were performed on Fortessa (BD Biosciences) and analyzed by FloJo software (Tree Star).

### Somatic hypermutation

The Peyer’s patches were excised from 8-week-old *ROD1* WT or KO mice and then gently dissociated by passage through 70 μm cell strainers. Germinal center B cells were double stained by anti-GL7-FITC (Biolegend, Catalog # 144603) and anti-Fas-APC (Biolegend, Catalog # 152603) antibodies, and were directly sorted into mouse genomic DNA extraction buffer by FACS Jazz (BD Biosciences). Genomic DNA was then purified following manufacturer’s protocol (Selleck, Catalog # B40013). JH4 intron was amplified by PCR using KOD DNA polymerase and a reported primer pair (F: 5′-GCCTGACATCTGAGGACTCTGC-3′ and R: 5′-CCTCTCCAGTTTCGGCTGAATCC-3′).^32^ JH4 amplicons were inserted into pCR-Blunt II-TOPO (Life technologies, Catalog # K280002) and sequenced with M13 primers by BGI. The mutation frequency was calculated from successfully sequenced clones by counting overall C/G or G/C mutations to total sequenced bases.

### Bone marrow transplantation in mice

Recipient mice were lethally irradiated (10 gray). *Ighm*^*tm1Cgn*^ (also known as muMT) mice were obtained from Jackson laboratory. CD45.1^+^/CD45.2^+^ mice were generated by crossing C57BL/6 mice with B6.SJL-Ptprc^a^ Pepc^b^/BoyJ mice. BM donor cells were isolated from either muMT, *ROD1*^−/−^, WT, B6 (CD45.2^+^), or B6.SJL (CD45.1^+^) mice. For muMT transplantation, BM cells from muMT and *ROD1*^−/−^ or WT mice were mixed at a ratio of 4:1, and 2× 10^6^ of total cells were transferred to a lethally irradiated host. Chimeric mice were rested in sterile cages for 6 weeks, and then BM and splenic B cells from chimeric mice were examined by FACS with diverse antibodies to B cell antigen receptor. For CD45.1^+^/CD45.2^+^ BMT, donor BM cells from CD45.1^+^ WT and CD45.2^+^ *ROD1*^−/−^ were equally mixed and transferred to lethally irradiated CD45.1^+^/CD45.2^+^ recipients. After 6 weeks, B cell subsets in BM and spleen were examined and quantified by FACS.

### Plasmid construction

The mouse ROD1 and AID expression vectors were purchased from OriGene (AID, Catalog # MR227286; ROD1, Catalog # MR208377). The AID and ROD1 coding region were amplified by PCR using KOD hot-start high fidelity DNA polymerase with primers shown in Table S5. HA-tagged AID and Flag-tagged ROD1 were inserted into pcDNA3.0 between HindIII and NotI. For bacterial protein expression, ROD1 and AID coding region were inserted into the pET-28a and pGEX-6p-1 plasmids, respectively. To make ROD1 knockout mice, three sgRNAs were synthesized (guide 1 # GTCGAGCTGTTCATGGTAGA, guide 2 # GAGAACACGGGAAGGCGAAC, guide 3 # GAGTCTTAAGTTCTCGGTGAT), annealed and ligated to the PX458 plasmid with the Bbs1 restriction site. To reconstitute AID and AID^147X^ mutant proteins in *AID*^−/−^ B cells, a 3× Flag tag sequence was first introduced into the N-terminus of AID and AID^147X^ by PCR amplification, the PCR products were then digested and inserted into pMX-IRES-GFP backbone between BamHI and EcoRI.

### Lentiviral shRNA packaging and transduction

The pLKO.1 lentiviral shRNA clones (TRCN0000306570, TRCN0000306569, TRCN00 00327080, and TRCN0000338854) were applied to knockdown ROD1. For lentiviral packaging, 6 μg of shRNA plasmid and packaging plasmids were co-transfected into 293T cells with Lipofectamine 2000 reagent. Virus was collected twice after 48 and 72 h of transfection. To promote transduction efficiency, 8 μg/ml of polybrene (Sigma, Catalog # H9268) was applied. The stably transduced cells were further selected by puromycin (2 μg/ml) for 3 days.

### Retrovirus packaging and transduction

Retroviral production and infection were performed as previously described.^60^ The pMX-3×Flag-mAID-IRES-GFP and pCL-ECO plasmids were co-transfected into ecotropic packaging cell lines (gift from Dr. Fei-long Meng) with Lipofectamine 2000. Retroviral supernatant was collected after 48 h transfection and passed through a 0.45 μm filter to remove any cell debris. Splenic B cells from 6–8 weeks old *AID*^−/−^ mice were prepared by negative selection and further stimulated by recombinant IL-4 and anti-CD40 antibody (eBioscience, Catalog # 16-0402-86) at a final concentration of 20 ng/ml and 1 μg/ml, respectively. For infection, 5 ml of virus-containing supernatant were mixed with 5 ml of pre-activated B cells in the presence of polybrene (16 μg/ml) and then incubated for 48–72 h at 37 °C.

### Immunofluorescence (IF)

For IF, splenic sections were blocked with 10% normal donkey serum in PBS for 1 h. After three times washing with PBS, the primary antibodies against B220 (1:50, eBioscience, Catalog # 12–0452), CD3 (1:30, eBioscience, Catalog # 50–0032) and ROD1 (1:30, Abnova, Catalog # H00009991-M01) were applied overnight at 4 °C. The sections were then incubated with donkey anti-mouse 488 (1:200; Invitrogen, Catalog # R37114) for 3 h at RT and counterstained with DAPI.

### Western blotting

Cells were lysed in 1× SDS sample buffer (50 mM Tris-HCl, pH 6.8, 2% SDS, 10% glycerol) without bromophenol blue and proteins were quantified by Nanodrop (Life Technologies). Fifty micrograms of total proteins was fractionated on a 10–12% SDS-polyacrylamide gel or 4–12% Bis-Tris protein gel (Life technology, Catalog # NP0322BOX) and transferred to a polyvinylidene difluoride membrane. The following antibodies were used: anti-ROD1 (1:1000, Abnova, Catalog # H00009991-M01; Proteintech, Catalog # 14027-1-AP), anti-GAPDH (1:8000, Cell Signaling Technology, Catalog # 2118L), anti-AID (1:500, Life Technologies, Catalog # 392500), anti-HA (1:500, Santa Cruz, Catalog # Sc-805) and anti-Flag (1:2000, Sigma, Catalog # F3165). Horseradish peroxidase (HRP)-coupled antibodies (1:5000) were purchased from Pierce (Catalog # 31460, 31430), and the light chain-specific secondary antibody mAb-HRP was from Cell Signaling Technology (Catalog # 5127S).

### Protein purification and pull-down assay

His-tagged proteins were purified with His GraviTrap column (GE Healthcare Life Sciences, Catalog # 11-0033-99). Briefly, plasmids were first transformed into the BL21 (DE3) strain, and single colonies were picked for overnight culture at 220 rpm/min. The cells were then diluted 1:20 with fresh LB medium and grown to an OD_600_ value of 0.6. To induce protein expression, IPTG was added to a final concentration of 1 mM and cultured for additional 2 h. Cells were collected by centrifuging at 4000 rpm for 30 min with a Thermo LYNX4000 super speed centrifuge. Cell pellets were resuspended in 50 ml of wash buffer (50 mM Na_2_HPO4, 1 M NaCl, 10 mM imidazole, pH 8.0, 1 mM PMSF) and disrupted by low temperature ultra-high pressure cell disrupters (JN-02C). The supernatant was then collected by centrifuging at 8000 × *g* for 30 min (Beckman Allegra 64 R) and directly applied to a His GraviTrap column, which was then washed 10 times with wash buffer I and three times with wash buffer II (50 mM Na_2_HPO4, 500 mM NaCl, 50 mM imidazole, pH 8.0) for getting rid of non-specific binding. After thoroughly washings, his-ROD1 proteins were eluted and concentrated to 3 ml with an ultra-centrifuge tube (Amicon, Catalog # UFC801096). For glutathione S-transferase (GST)-tagged protein purification, GST beads (GE Healthcare Life Sciences, catalog # 17-0756-01) were used to capture bacterially expressed proteins from the supernatant and washed twice with high salt buffer (10 mM Tris-HCl, 1 M NaCl, 0.5 mM EDTA, 0.1% NP-40, pH 8.0). The beads were then washed twice with PBS buffer and finally eluted with 1.5 ml of elution buffer containing 10 mM GSH (Amresco, catalog # 70-18-8). For pull-down assay, either AID or ROD1 was immobilized to beads and then used as a bait to pull down interacting proteins in 1× PBS buffer for 1 h. After that, the proteins were eluted from the beads with 1× SDS loading buffer and fractionated by 4–12% SDS-PAGE gels. To visualize protein bands, Coomassie blue G-250 (Urchem, catalog # 71011381) was used for staining.

### Crosslinking and Immunoprecipitation sequencing (CLIP-seq)

CLIP-seq was performed as previously described.^61^ Briefly, the 2× 10^8^ isolated B cells were irradiated at 400 mJ/cm^2^ with 254 nm UV light. The cross-linked cells were lysed and 15 μg of anti-ROD1 or anti-AID antibody was applied to pull down specific protein-RNA complexes. After micrococcal nuclease treatment, 3′ RNA linker ligation and 5′ end ^32^P labeling, the immunoprecipitated complexes were fractionated on a 4–12% NuPAGE Bis-Tris gel and transferred to a nitrocellulose membrane. The autoradiographed ROD1- or AID-specific smear bands were cut and treated with proteinase K (Takara, Catalog # 9034) prior to the extraction of respective RNA by phenol and chloroform. A 5′ RNA linker was then added to the isolated RNA and reverse transcribed by superscript reverse transcriptase III (Life Technologies, catalog # 18080–051). After PCR amplification and deep-sequencing, the sequenced reads were first trimmed by removing the 5′-adaptor and 3′-adaptor sequences, and then mapped to the mouse reference genome (mm9) with Bowtie2^62^; two mismatches were allowed for mapping. CLIP-seq peaks were identified by Piranha version 1.2.1 with the following parameters: -s -b 20 -d ZeroTruncatedNegativeBinomial -p 1e-5 or 2e-3.^63^ Binding motifs were deduced by the MEME software^64^ based on the default parameters. Meta-profiles were generated with the program ngs.plot.^65^

### RNA immunoprecipitation and quantitative PCR (RIP-qPCR)

To transiently block transcription, 5,6-dichlorobenzimidazole 1-β-D-ribofuranoside (Sigma, Catalog # D1916, final concentration of 100 µM) or flavopiridol (Sigma, Catalog # F3055, final concentration of 40 nM) was added to LPS-activated B cells for 4 h. 1× 10^8^ of treated cells were washed twice with PBS and centrifuged at 400 × *g* for 5 min. The cell pellets were resuspended in 15 ml of ice-cold PBS and cross-linked by UV light at 254 nm with energy of 400 mJ/cm^2^. The cross-linked cells were collected and resuspended in 1 ml of RIP buffer (50 mM Tris-HCl pH 7.5, 150 mM NaCl, 1% NP40, 0.5% Sodium deoxycholate, 1 mM PMSF, 1× protease inhibitor cocktail) and lysed on ice for 15 min. The cell lysate was further treated by sonication with BRANSON SLPe (output setting 4, 10 s per cycle) for 10 times. The cell debris and pellets were removed by centrifuging at 13,000 rpm at 4 ℃ for 10 min. The supernatant was pre-cleared for 30 min by adding 15 μl protein A/G beads (Pierce, Catalog # 88803) and 20 μg/ml of yeast tRNA. To each reaction, 6 μg of antibodies were first coupled to 20 μl protein A/G beads and then added to pre-cleared supernatant. After 4 h, the beads were sequentially washed three times with washing buffer I (50 mM Tris-HCl pH 7.5, 1 M NaCl, 1% NP40, 1% Sodium Deoxycholate) and washing buffer II (50 mM Tris-HCl pH 7.5, 1 M NaCl, 1% NP40, 1% Sodium Deoxycholate, 1 M urea). The immunoprecipitated products were eluted three times with elution buffer (100 mM Tris-HCl pH 7.0, 5 mM EDTA, 10 mM DTT, 1% SDS). To digest away proteins, 5 μl of proteinase K was added to the elution buffer and incubated at 55 ℃ for 30 min. RNA was extracted by phenol/chloroform extraction and then precipitated by ethanol with 1 μl glycogen (Life technologies, Catalog # AM9515) as carrier. The RNA pellet was resuspended in 15 μl H_2_O and reverse transcribed with random primer.

### 3D protein structure docking

To model the interaction surface between ROD1 and AID, mouse AID (UniProt entry Q9WVE0) and ROD1 (UniProt entry Q8BHD7) were first predicted by an interactome 3D server at http://interactome3d.irbbarcelona.org/. The PDB ID/model ID of 1QM9 and 3V4K were selected and downloaded for ROD1 and AID, respectively. Then, the protein-protein interaction was docked and modeled by using the PRISM 2.0 server at http://cosbi.ku.edu.tr/prism. The docking structure with the lowest energy was further visualized by PyMol (V1.7.2.1, http://www.pymol.org/).

### TC-seq data analysis

Genome-wide translocation capture sequencing data were downloaded from the Sequenced Read Archive (SRA) with the accession number SRA039959.^42^ The analysis was performed using a previously described computational workflow and aligned to the mouse genome by BWA.^66^

### Chromatin immunoprecipitation combined with quantitative PCR (ChIP-qPCR)

B cells and CH12F3 cells were cross-linked with 1% formaldehyde for 10 min at RT. Fixation was stopped by 125 mM glycine for 5 min and the samples were washed twice with ice-cold PBS. Cell pellets were then resuspended in 1 ml of cyto lysis buffer, mixed briefly and incubated on ice for 10–15 min with occasional inversion every 2 min. Cells were then centrifuged for 5 min at 3500 rpm at 4 °C, the supernatant was discarded, and the remaining nuclear pellet (white) was resuspended in 500 μl of nuclear lysis buffer (1% SDS, 10 mM EDTA, 50 mM Tris-Cl, pH 8.1, 1× protease inhibitor cocktail). The samples were then sonicated seven times at the maximum setting (BRANSON SLPe, output setting 4, 10 sec per cycle). The soluble chromatin was collected by centrifuging for 10 min at 14,000 rpm, and the supernatant was diluted 1:10 with dilution buffer (150 mM NaCl, 20 mM Tris-HCl, pH 8.1, 2 mM EDTA, 1% Triton X-100 and 1× protease inhibitor cocktail). Chromatin was incubated at 4 °C overnight with protein A/G beads that pre-coupled with anti-AID antibody. Anti-mouse IgG (Santa Cruz, Catalog # Sc-2762) was used as a negative control. The precipitated AID complexes were washed twice in low-salt buffer (150 mM NaCl, 20 mM Tris-HCl, pH 8.1, 2 mM EDTA, 1% Triton X-100, 0.1% SDS), twice in high-salt buffer (500 mM NaCl, 20 mM Tris-HCl, pH 8.1, 2 mM EDTA, 1% Triton X-100, 0.1% SDS), twice in LiCl buffer (250 mM LiCl, 1% NP-40, 1% deoxycholate, 1 mM EDTA, pH8.0, 10 mM Tris-HCl, pH 8.1) and twice in TE buffer (pH 8.0). The thoroughly washed beads were eluted twice with 150 μl of elution buffer (0.1 M NaHCO_3_, 1% SDS) by vortexing at 70 °C at 1000 rpm for 10 min on a Thermo Mixer C (Eppendorf). The enriched DNA fragments were then purified with Qiaquick spin column and quantified by Nanodrop. The published primers of *Cd83*, *Cd79b*, *c-myc*, *Pax5*, and *Pim1*^32^ were used for AID occupancy analysis with SYBR green master mix (Roche, Catalog # 17747200).

### Global run-on sequencing (Gro-seq)

Gro-seq was performed as previously described.^67^ Briefly, Splenic B cells from 8-week-old *ROD1* WT and KO mice were ex vivo cultured and stimulated with LPS for 3 days. Nuclei was isolated with ice-cold swelling buffer (10 mM Tris-HCl, pH 7.5, 2 mM MgCl_2_, 3 mM CaCl_2_) and stored in freezing Buffer (40 % Glycerol, 5 mM MgCl_2_, 0.1 mM EDTA, 50 mM Tris-HCl, pH8.3, 2 U/ml RNaseOut). 5× 10^7^ nuclei were mixed with an equal volume of reaction buffer (10 mM Tris-Cl pH 8.0, 5 mM MgCl_2_, 1 mM DTT, 300 mM KCl, 200 U/ml RNaseOut, 1% sarkosyl, 500 μM ATP, GTP, Br-UTP and 2 µM CTP) and incubated at 30 °C for 5 min. The RNA was extracted by Phenol/chloroform and precipitated by ethanol. The pellet was resuspended in 20 μl of DEPC-treated water. RNA was then fragmented and purified with anti-BrdU beads (Santa Cruz Biotech, Catalog # Sc-32323AC). The BrdU enriched RNA was polyA-tailed using E.coli Poly (A) polymerase and then reverse transcribed. After circulation and relinearization by APE1 (15 U; New England Biolabs, Catalog # M0282S), the cDNA was PCR amplified and gel purified for sequencing by Illumina HiSeq 2500.

### Statistical analysis

The data are presented as the means and standard deviations. Two-tailed Student’s *t* test was used to calculate the differences between two groups. The degree of freedoms was defined as the number of group 1 plus group 2 minus two. The significance of overlap among AID/ROD1 targets, chromosomal translocation hotspots and hypermutation targets was calculated by Fisher’s exact test. Pearson’s correlation coefficients (*R*) were calculated by using reads number in 10-kb-binned regions of the whole genome. Kolmogorov–Smirnov test was used to compare the distributions of CLIP-seq signal for two sets of genes. The Wilcoxon rank-sum test was applied to calculate the ROD1 or AID CLIP-seq binding difference among translocation hotspot genes and the remaining genes in LPS-activated B cells. A value of *P* < 0.05 was considered to be statistically significant.

### Accession number

Gene Expression Omnibus: GSE94662.

## Electronic supplementary material

Supplementary information, Figure S1

Supplementary information, Figure S2

Supplementary information, Figure S3

Supplementary information, Figure S4

Supplementary information, Figure S5

Supplementary information, Figure S6

Supplementary information, Figure S7

Supplementary information, Figure S8

Supplementary information, Figure S9

Supplementary information, Figure S10

Supplementary information, Figure S11

Supplementary information, Figure S12

Supplementary information, Figure S13

Supplementary information, Figure S14

Supplementary information, Figure S15

Supplementary information, Figure S16

Supplementary information, Figure S17

Supplementary information, Figure S18

Supplementary information, Figure S19

Supplementary information, Table S1

Supplementary information, Table S2

Supplementary information, Table S3

Supplementary information, Table S4

Supplementary information, Table S5

## Electronic supplementary material

**Supplementary information** accompanies for this paper at 10.1038/s41422-018-0076-9.
