# Supplementary material for: The RNA-binding protein ROD1/PTBP3 cotranscriptionally defines AID-loading sites to mediate antibody class switch in mammalian genomes
Source: Cell Res. 2018 Aug 24;28(10):981–95. doi: 10.1038/s41422-018-0076-9 (PMC6170407; doi:10.1038/s41422-018-0076-9)
Supplement: Supplementary file 1 — Supplementary information, Figure S1 [file 41422_2018_76_MOESM1_ESM.pdf]

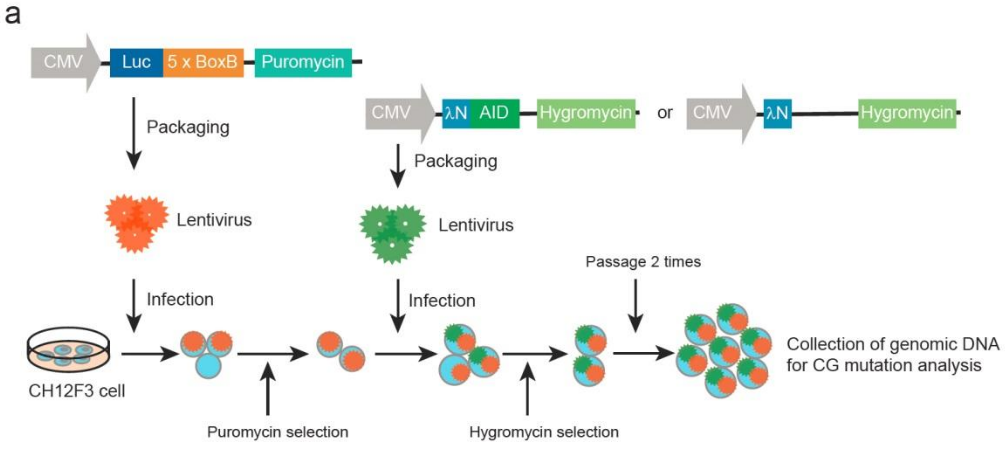

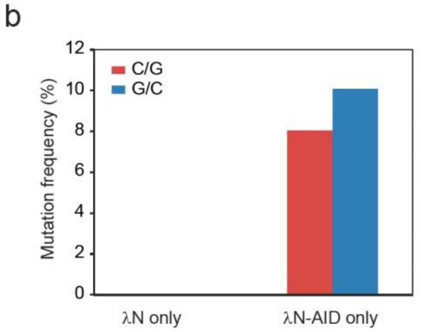

c

	λN only	λN-AID
Total clone analyzed	15	15
Total base analyzed	3525	3525
Number of mutations	2	1041
Number of C->G mutations	0	71
Number of G->C mutations	0	92
Number of AT mutations	0	158
Total mutation frequency/bp	0.000567	0.295
C->G mutation frequency/C	0	0.08
G->C mutation frequency/G	0	0.101
AT mutation frequency/AT	0	0.092

Supplementary Figure 1. λN/BoxB tethering assay in stably transduced CH12F3 cells. (**a**) The stable cell lines were generated by the sequential transduction of 5× BoxB-reporter and λN-AID or λN alone. The CH12F3 cells were first infected by lentivirus containing the expression unit of 5× BoxB-reporter, after puromycin selection for 2 days, the lentiviral form of λN-AID or λN alone was further applied and selected with hygromycin for another 3 days. The two selected cell lines were further passaged twice and then genomic DNA was collected for PCR amplification and Sanger sequencing. (**b**) The mutation frequency observed in the BoxB region upon transduction with λN alone or λN-AID. The mutation frequency was calculated from 15 sequenced clones. (**c**) Summary of the sequenced clones and mutation spectrum as shown in (b).
