# Supplementary material for: The RNA-binding protein ROD1/PTBP3 cotranscriptionally defines AID-loading sites to mediate antibody class switch in mammalian genomes
Source: Cell Res. 2018 Aug 24;28(10):981–95. doi: 10.1038/s41422-018-0076-9 (PMC6170407; doi:10.1038/s41422-018-0076-9)
Supplement: Supplementary file 2 — Supplementary information, Figure S2 [file 41422_2018_76_MOESM2_ESM.pdf]

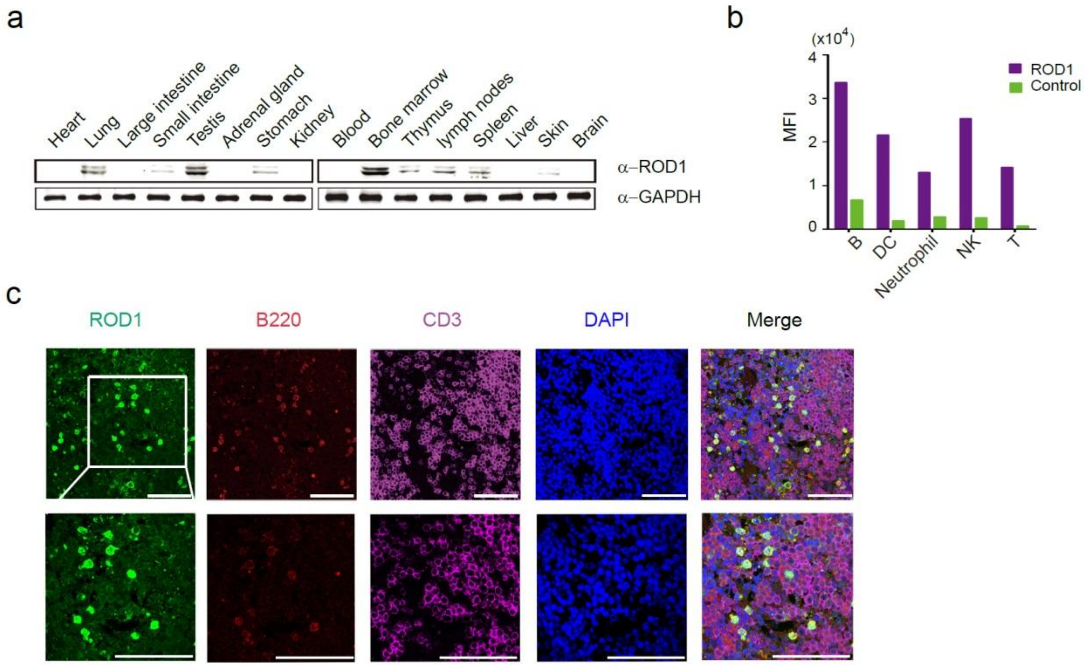

Supplementary Figure 2. ROD1 is highly expressed in B cells. (**a**) ROD1 expression in lysates extracted from diverse mouse tissues. GAPDH served as a loading control. (**b**) Flow cytometric analysis of ROD1 expression in isolated lymphoid cells, dendritic cells and neutrophils from spleen. MFI: mean fluorescence intensity. (**c**) Immunofluorescence staining of ROD1, B220 and CD3 in splenic sections. ROD1 is in green, B220 in red, CD3 in purple and DAPI in blue. Scale bar: 50 μm.
