# Supplementary material for: The RNA-binding protein ROD1/PTBP3 cotranscriptionally defines AID-loading sites to mediate antibody class switch in mammalian genomes
Source: Cell Res. 2018 Aug 24;28(10):981–95. doi: 10.1038/s41422-018-0076-9 (PMC6170407; doi:10.1038/s41422-018-0076-9)
Supplement: Supplementary file 4 — Supplementary information, Figure S4 [file 41422_2018_76_MOESM4_ESM.pdf]

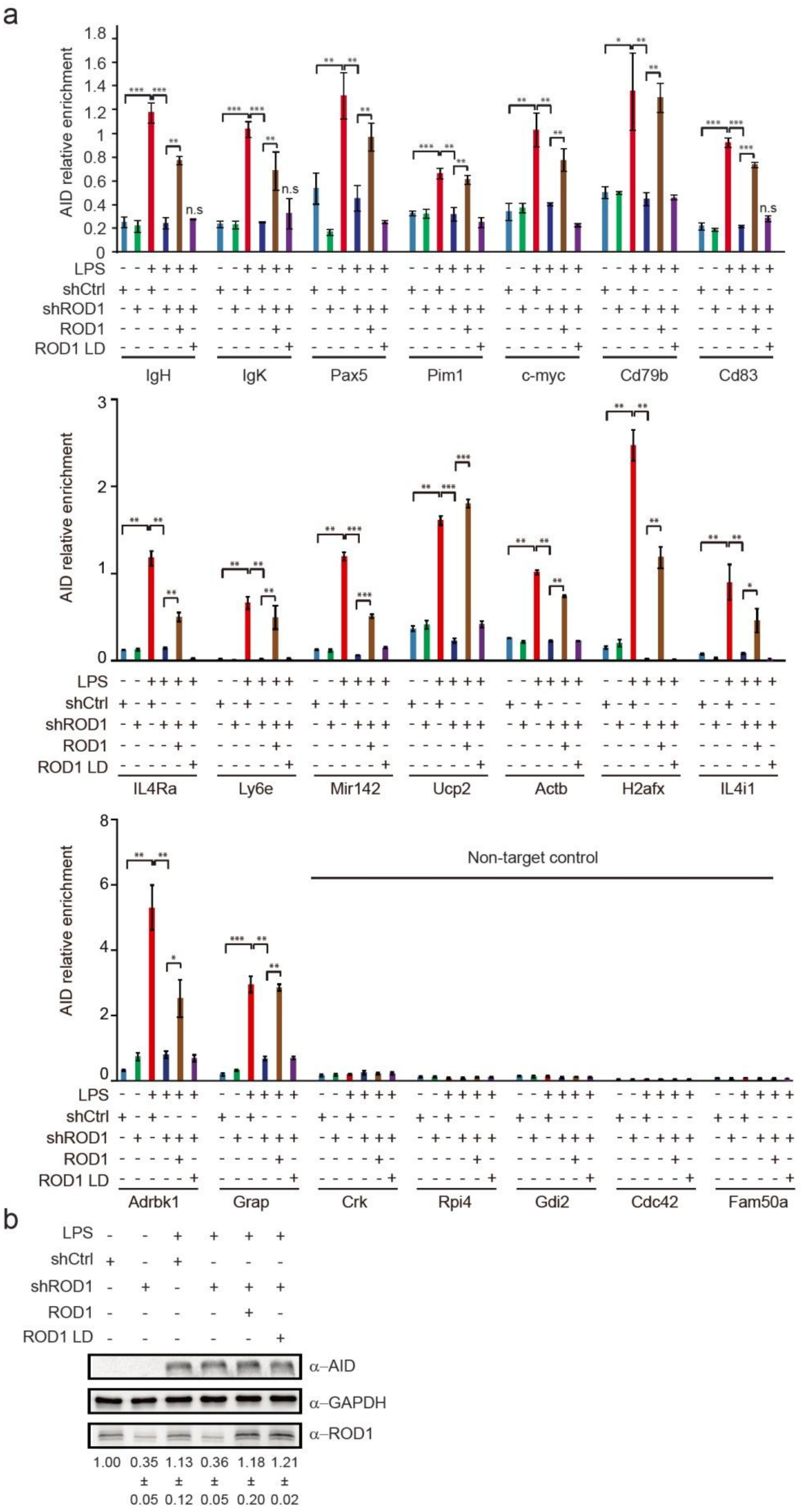

Supplementary Figure 4. The loop region is required for AID targeting. (**a**) ChIP-qPCR for AID occupancy in *Ig* and non-*Ig* targets of primary B cells infected with shROD1,

shCtrl, ROD1 and a ROD1 loop deletion mutant (LD). ChIP was performed with either anti-AID or control IgG. Five AID non-targets served as negative controls. *$P<0.05$, **$P<0.01$, ***$P<0.001$ and $P>0.05$ was non-significant (n.s.) by two-tailed Student's *t*-test. Data are shown as the mean ± SD (n = 3) and normalized against input DNA. (**b**) Western blotting of ROD1 and AID expression in differently treated B cells. A shRNA-resistant mutant with same protein coding potential was applied for the rescue experiment. GAPDH served as a loading control. ROD1 expression levels were quantified and shown at the bottom. Data are shown as the mean ± SD (n = 3).
