# Supplementary material for: The RNA-binding protein ROD1/PTBP3 cotranscriptionally defines AID-loading sites to mediate antibody class switch in mammalian genomes
Source: Cell Res. 2018 Aug 24;28(10):981–95. doi: 10.1038/s41422-018-0076-9 (PMC6170407; doi:10.1038/s41422-018-0076-9)
Supplement: Supplementary file 5 — Supplementary information, Figure S5 [file 41422_2018_76_MOESM5_ESM.pdf]

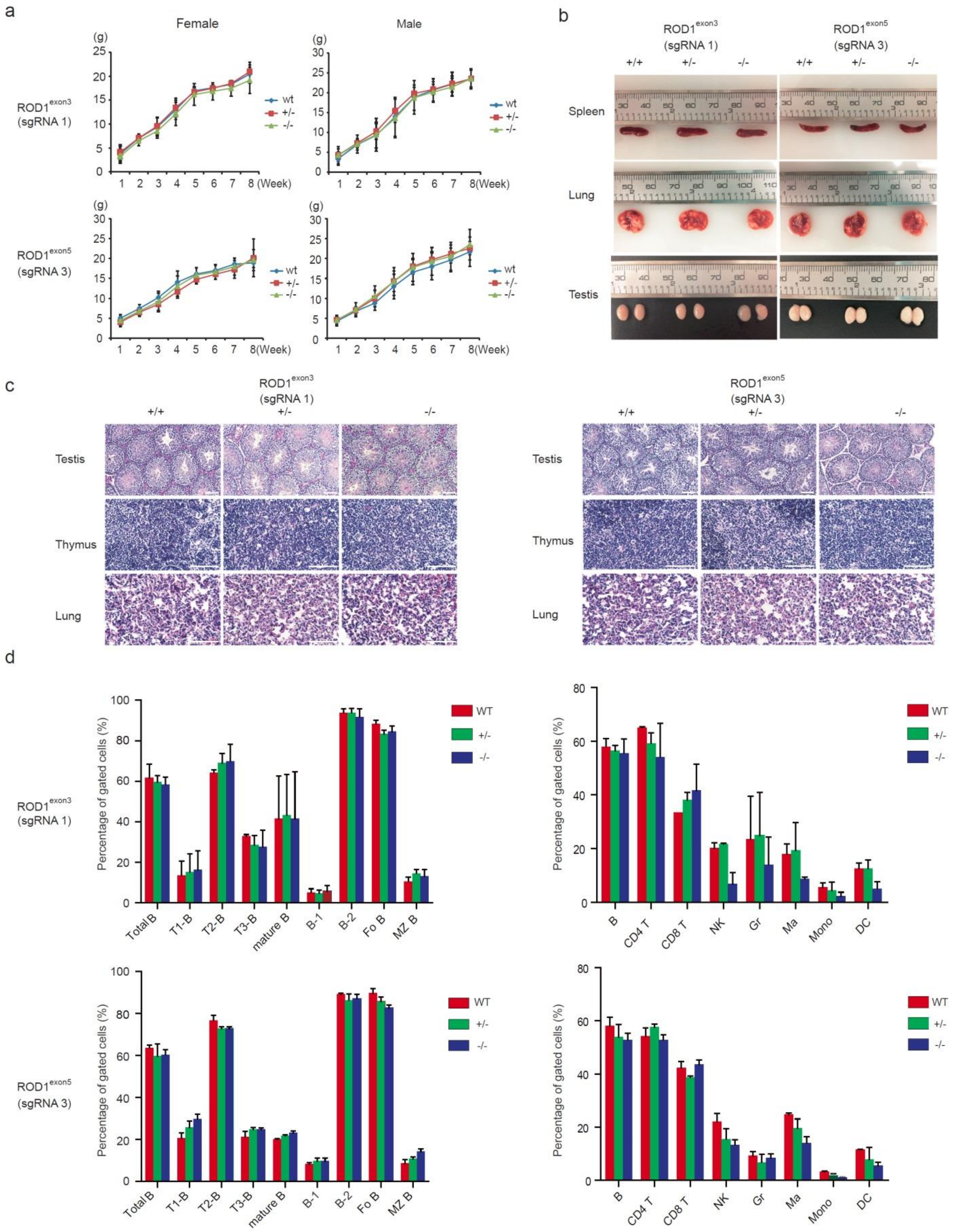

Supplementary Figure 5. ROD1 knockout mice are normal and fertile. (**a**) The body weights recorded for different ROD1 genotypes and sexes at different time points. ROD1 knockout mice were generated with either sgRNA 1 targeting exon 3 or sgRNA 3

targeting exon 5. For each genotype and sex, 10 age-matched mice were measured from week 1 to week 8. (**b**) Representative morphology of the spleen, lung and testis from 8-week-old *ROD1*$^{+/+}$, *ROD1*$^{+/-}$ and *ROD1*$^{-/-}$ mice. N = 3 for each genotype. (**c**) Hematoxylin and eosin staining of testis, thymus and lung sections from 8-week-old mice (n = 3). Scale bar: 50 μm. (**d**) Flow cytometric analysis of splenic lymphocyte numbers in different genotypes (n = 3). Fo B: Follicular B; MZ B: Marginal zone B; NK: Natural killer; Gr: Granular; Ma: Macrophage; Mono: Monocyte; DC: Dendritic cell; Pro B: Progenitor B; Pre B: Precursor B.
