# Supplementary material for: The RNA-binding protein ROD1/PTBP3 cotranscriptionally defines AID-loading sites to mediate antibody class switch in mammalian genomes
Source: Cell Res. 2018 Aug 24;28(10):981–95. doi: 10.1038/s41422-018-0076-9 (PMC6170407; doi:10.1038/s41422-018-0076-9)
Supplement: Supplementary file 6 — Supplementary information, Figure S6 [file 41422_2018_76_MOESM6_ESM.pdf]

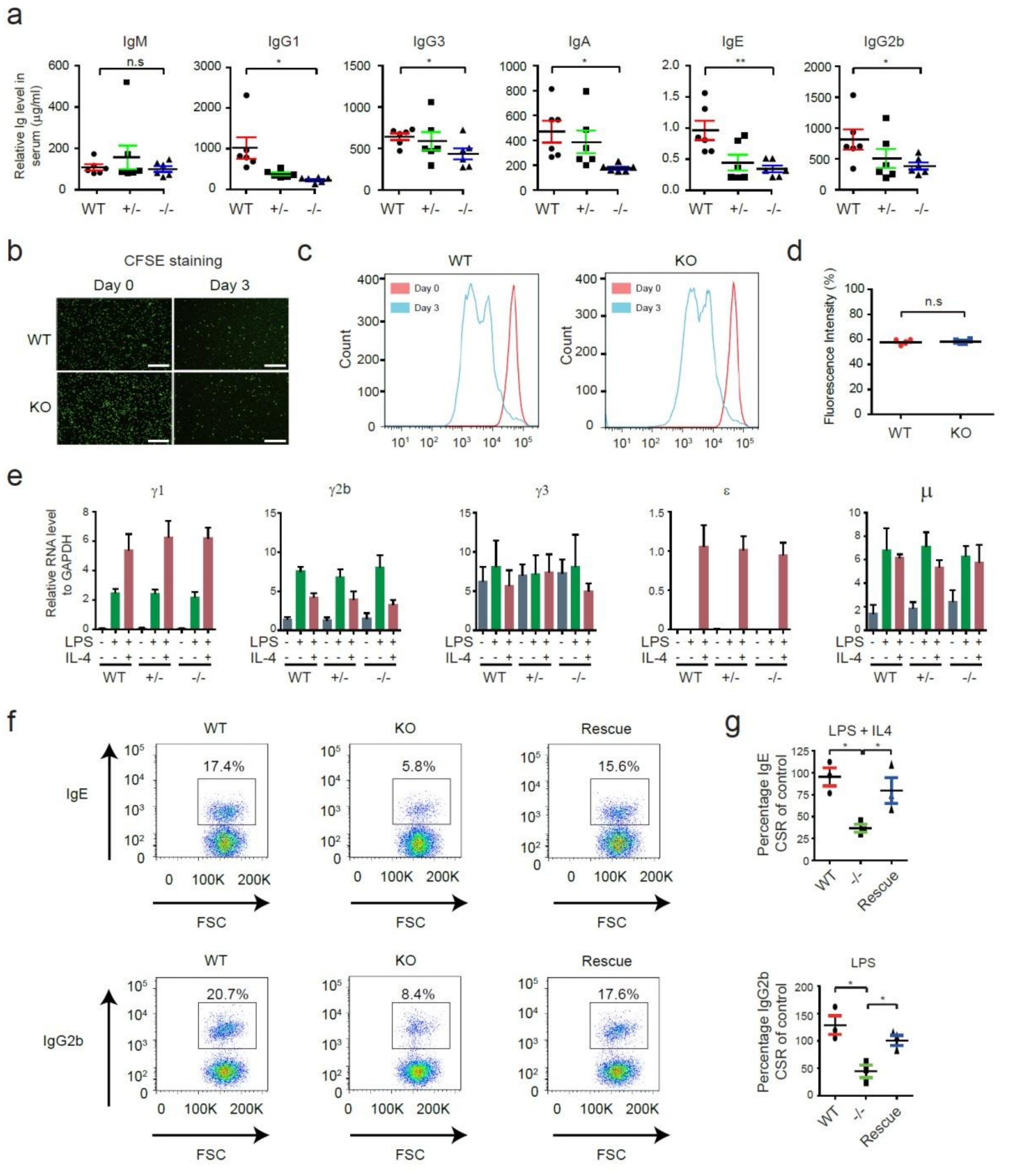

Supplementary Figure 6. CSR defects in *ROD1*$^{exon3-/-}$ mice. (**a**) The Ig concentration was quantified by ELISA with blood from WT and *ROD1*$^{exon3-/-}$ mice. Blood samples were harvested from 8-week-old mice (n = 6 for each genotype). **P*<0.05, ***P*<0.01 and *P*>0.05 was non-significant (n.s) as determined by two-tailed Student's *t*-test. (**b**) CFSE staining of splenic B cells before (day 0) and after LPS stimulation (day 3). B cells were isolated from age-matched 8-week-old WT and *ROD1*$^{exon3-/-}$ mice. Scale bar: 50 μm. (**c**) Flow cytometric analysis of splenic B cells stained with CFSE before (red) and after (blue) LPS treatment. A portion of the B cells was harvested and measured at day 0. The rest of the population was stimulated with LPS and allowed to proliferate for 3 days. (**d**) The quantification of B cell proliferation for WT and *ROD1*$^{exon3-/-}$ mice. The y-axis represents the intensity of CFSE dye (n = 4). (**e**) RT-qPCR examination of germline transcripts containing Iμ and C_H exons of the same isotype in *ex vivo* cultured splenic B

cells. (**f**) Flow cytometric analysis of IgE and IgG2b with *ex vivo* cultured splenic B cells activated either by LPS or LPS plus IL4. (**g**) Quantification of IgE and IgG2b CSR efficiency as shown in (f). $^*P<0.05$ by two-tailed Student's *t*-test. (f) and (g) are related to Fig. 2c.
