# Supplementary material for: The RNA-binding protein ROD1/PTBP3 cotranscriptionally defines AID-loading sites to mediate antibody class switch in mammalian genomes
Source: Cell Res. 2018 Aug 24;28(10):981–95. doi: 10.1038/s41422-018-0076-9 (PMC6170407; doi:10.1038/s41422-018-0076-9)
Supplement: Supplementary file 7 — Supplementary information, Figure S7 [file 41422_2018_76_MOESM7_ESM.pdf]

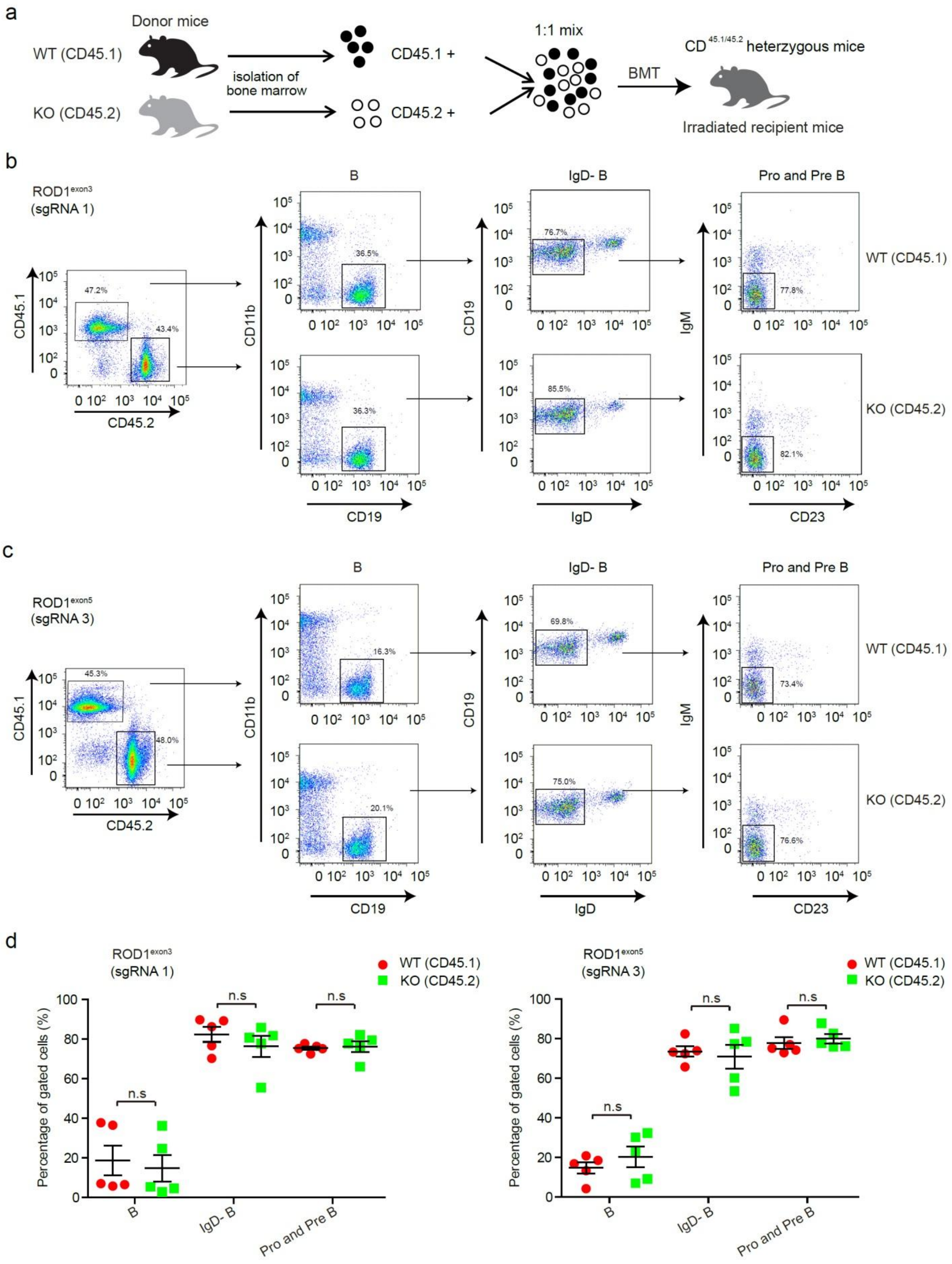
a
Donor mice
WT (CD45.1)
KO (CD45.2)
isolation of bone marrow
CD45.1 +
CD45.2 +
1:1 mix
BMT
CD 45.1/45.2 heterzygous mice
Irradiated recipient mice
b
ROD1exon3 (sgRNA 1)
c
ROD1exon5 (sgRNA 3)
B
IgD- B
Pro and Pre B
CD45.1
CD45.2
CD11b
CD19
IgD
IgM
CD23
WT (CD45.1)
KO (CD45.2)
47.2%
43.4%
36.5%
36.3%
76.7%
85.5%
77.8%
82.1%
45.3%
48.0%
16.3%
20.1%
69.8%
75.0%
73.4%
76.6%
d
ROD1exon3 (sgRNA 1)
ROD1exon5 (sgRNA 3)
WT (CD45.1)
KO (CD45.2)
Percentage of gated cells (%)
n.s
B
IgD- B
Pro and Pre B

Supplementary Figure 7. ROD1 depletion has no influence on B cell differentiation in the bone marrow of CD45.1 and CD45.2 mixed chimeras. (**a**) Generation of chimeric mice by transplantation of mixed bone marrow cells from *ROD1*$^{-/-}$ (CD45.2) and WT (CD45.1) donor mice into irradiated CD45.1^{+}/45.2^{+} recipients. (**b**, **c**) Flow cytometric analysis of repopulating bone marrow cells in WT and *ROD1*$^{-/-}$ chimeric mice after 6 weeks of reconstitution (n = 5). Numbers on plots indicate percentages of plotted cells in the gate (axes are in arbitrary units of fluorescence intensity). The cell gating strategy is shown by the thin arrowhead. Pro B: Progenitor B; Pre B: Precursor B. (**d**) Quantification of B cell subsets in the bone marrows from WT and *ROD1*$^{-/-}$ chimeric mice as shown in (b, c). Data are shown as the mean ± SD (n = 5). The n.s represents non-significant, two-tailed Student's *t*-test.
