# Supplementary material for: The RNA-binding protein ROD1/PTBP3 cotranscriptionally defines AID-loading sites to mediate antibody class switch in mammalian genomes
Source: Cell Res. 2018 Aug 24;28(10):981–95. doi: 10.1038/s41422-018-0076-9 (PMC6170407; doi:10.1038/s41422-018-0076-9)
Supplement: Supplementary file 8 — Supplementary information, Figure S8 [file 41422_2018_76_MOESM8_ESM.pdf]

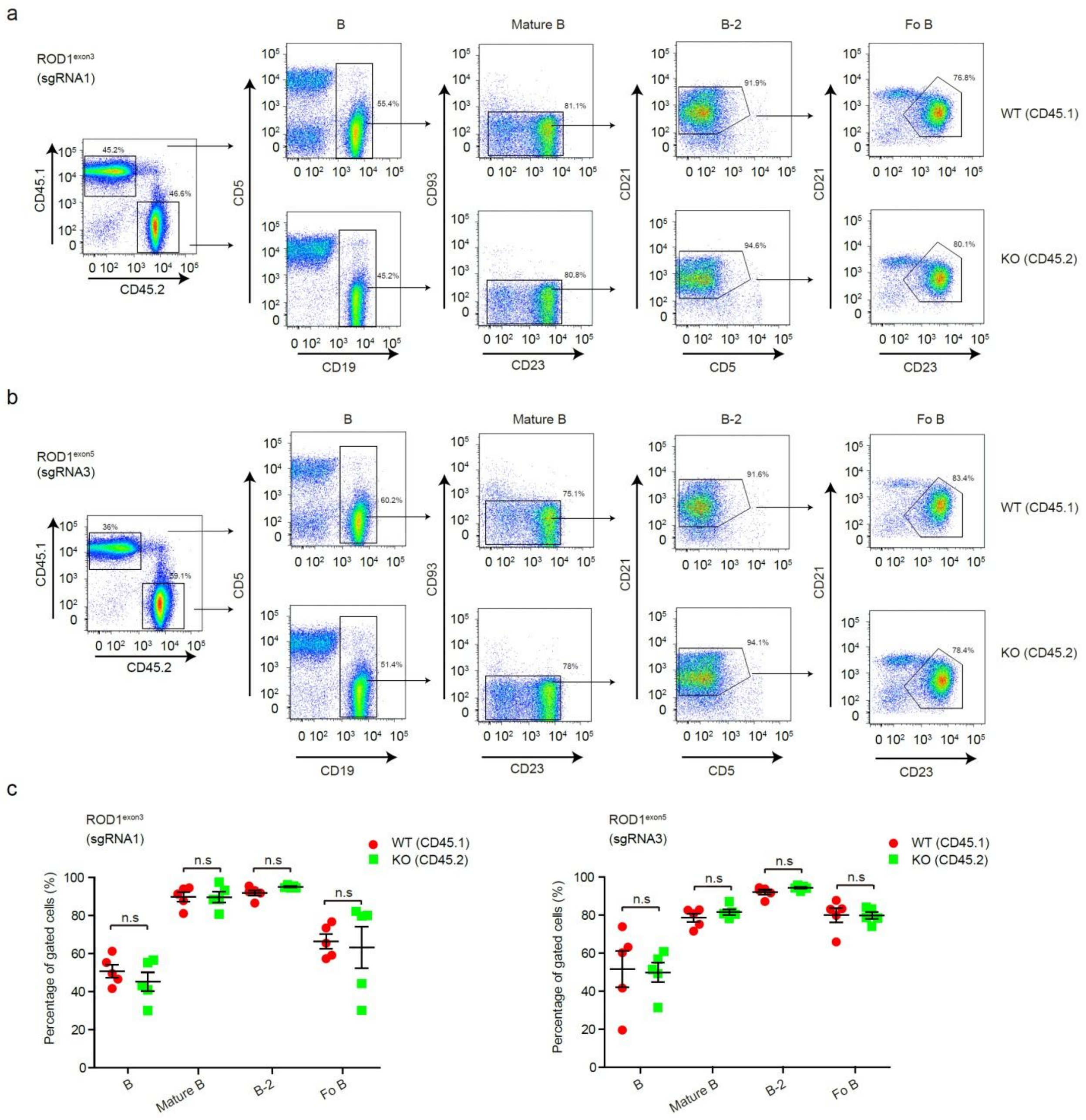

Supplementary Figure 8. ROD1 ablation has no influence on B cell differentiation in the spleen of CD45.1 and CD45.2 mixed chimeras mice**.** (**a**, **b**) Flow cytometric analysis of the repopulating splenic B cells in WT and *ROD1*$^{-/-}$ chimeric mice after 6 weeks of reconstitution (n = 5). Fo B: Follicular B. (**c**) Quantification of B cell subsets in the spleens of WT and *ROD1*$^{-/-}$ chimeric mice as shown in (a, b). Data are shown as the mean ± SD (n = 5). The n.s represents non-significant, two-tailed Student's *t*-test.
