# Supplementary material for: The RNA-binding protein ROD1/PTBP3 cotranscriptionally defines AID-loading sites to mediate antibody class switch in mammalian genomes
Source: Cell Res. 2018 Aug 24;28(10):981–95. doi: 10.1038/s41422-018-0076-9 (PMC6170407; doi:10.1038/s41422-018-0076-9)
Supplement: Supplementary file 9 — Supplementary information, Figure S9 [file 41422_2018_76_MOESM9_ESM.pdf]

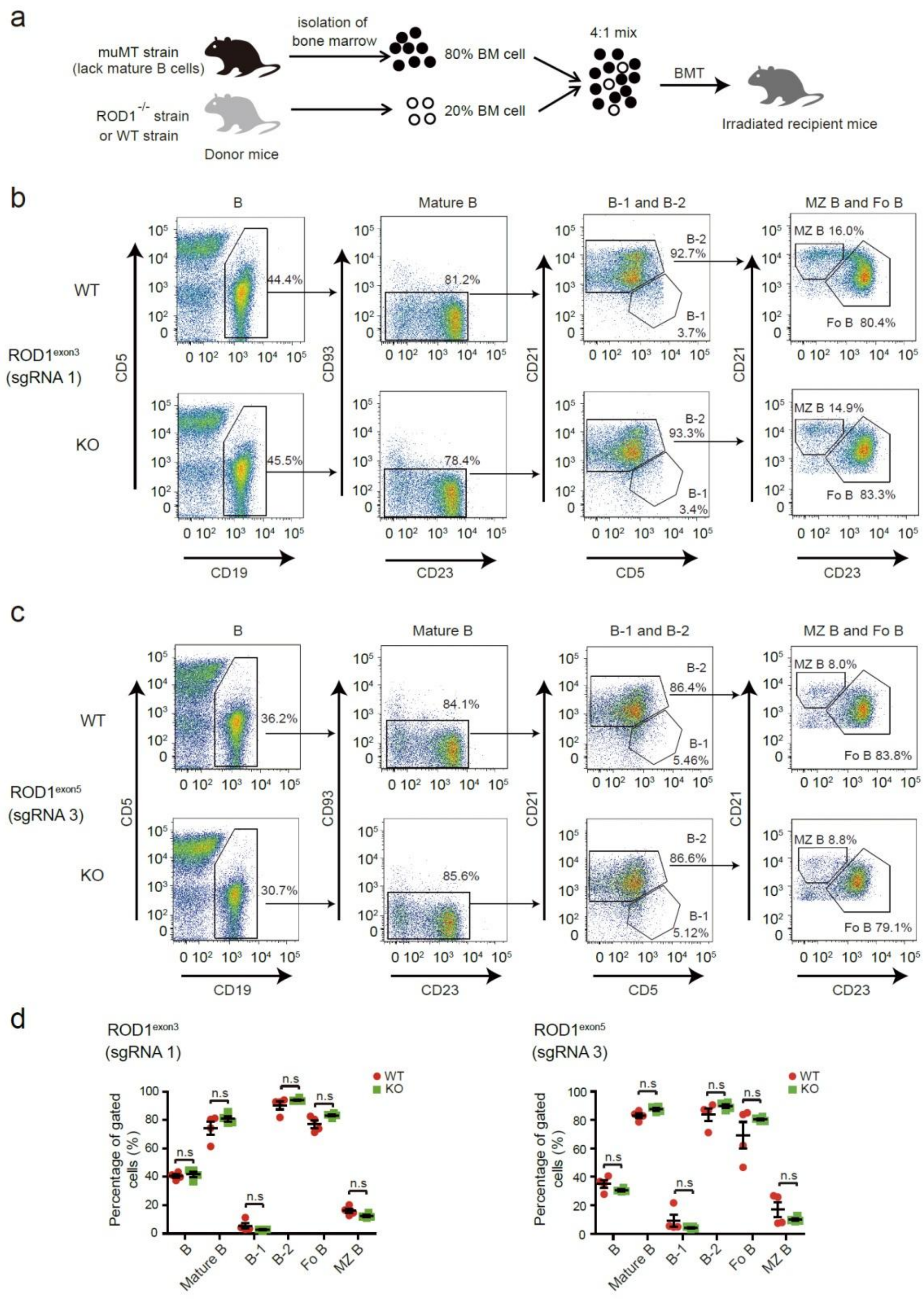

Supplementary Figure 9. The quantification of repopulating splenic B cells in muMT and WT or *ROD1*$^{-/-}$ chimeric mice. (**a**) Generation of bone marrow chimeric mice where donors and/or recipients were either muMT, *ROD1*$^{-/-}$ or WT mice. (**b**, **c**) Flow cytometric analysis of repopulating splenic B cells in muMT and WT or *ROD1* $^{-/-}$ chimeric mice (n = 4). Numbers on plots indicate percentages of plotted cells in the gate. The cell gating strategy is shown by the thin arrowhead. Fo B: Follicular B; MZ B: Marginal zone B. (**d**)

Quantification of B cell subsets in spleens of muMT and WT or *ROD1* $^{-/-}$ chimeric mice as shown in (b, c). The data are presented as the mean ± SD.
