# Supplementary material for: The RNA-binding protein ROD1/PTBP3 cotranscriptionally defines AID-loading sites to mediate antibody class switch in mammalian genomes
Source: Cell Res. 2018 Aug 24;28(10):981–95. doi: 10.1038/s41422-018-0076-9 (PMC6170407; doi:10.1038/s41422-018-0076-9)
Supplement: Supplementary file 10 — Supplementary information, Figure S10 [file 41422_2018_76_MOESM10_ESM.pdf]

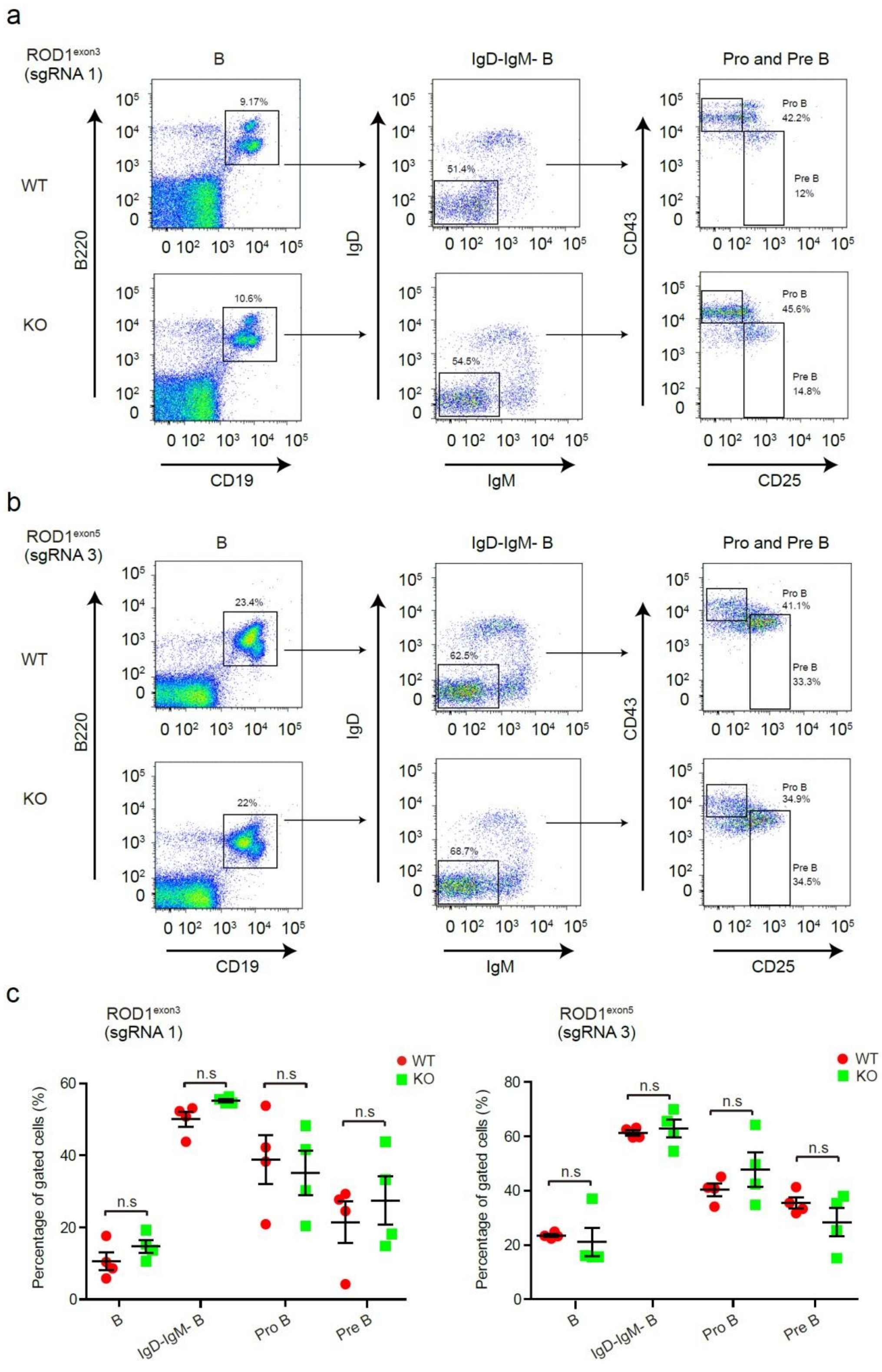

Supplementary Figure 10. ROD1 ablation has no influence on B cell differentiation in the bone marrow of muMT chimeric mice. (**a**, **b**) Flow cytometric analysis of repopulating bone marrow B cells in muMT and WT or *ROD1*$^{-/-}$ chimeric mice after 6

weeks of transplantation (n = 4). Pro B: Progenitor B; Pre B: Precursor B. (**c**) Quantification of B cell subsets in the bone marrows of muMT and WT or *ROD1*$^{-/-}$ chimeric mice (n = 4).
