# Supplementary material for: The RNA-binding protein ROD1/PTBP3 cotranscriptionally defines AID-loading sites to mediate antibody class switch in mammalian genomes
Source: Cell Res. 2018 Aug 24;28(10):981–95. doi: 10.1038/s41422-018-0076-9 (PMC6170407; doi:10.1038/s41422-018-0076-9)
Supplement: Supplementary file 11 — Supplementary information, Figure S11 [file 41422_2018_76_MOESM11_ESM.pdf]

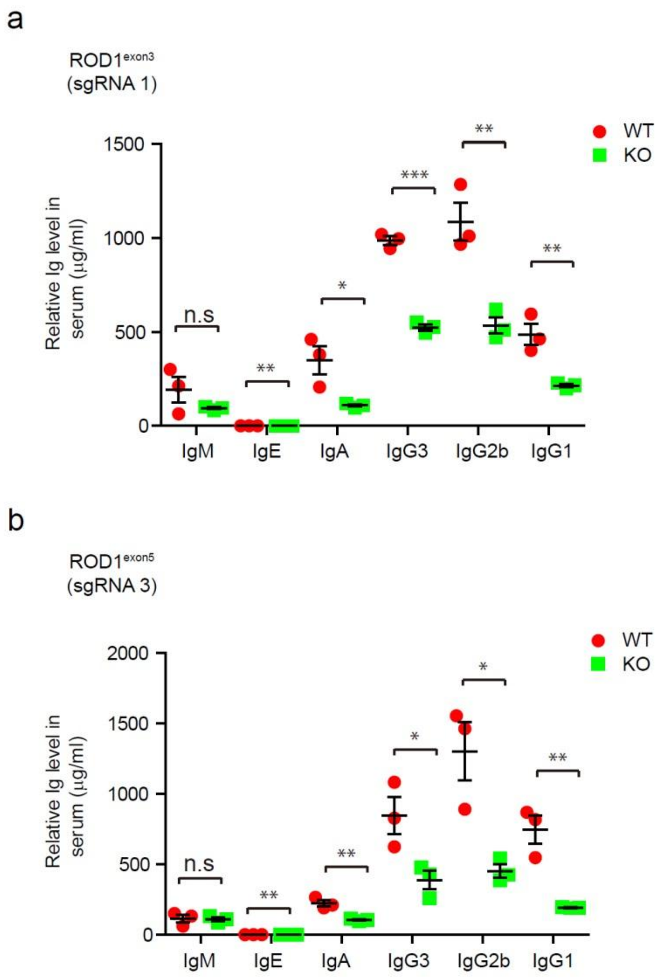

Supplementary Figure 11. ROD1 ablation causes reduced antibody titers in the muMT and *ROD1*$^{-/-}$chimeric mice. (**a**, **b**) The Ig concentration was quantified by ELISA with blood from muMT and WT or *ROD1*$^{-/-}$ chimeric mice, respectively. Blood samples were harvested after 6 weeks of mixed bone marrow reconstitution (n = 3 for each genotype). *P<0.05, **P<0.01, ***P<0.001 and P>0.05 was non-significant (n.s) as determined by two-tailed Student's *t*-test.
