# Supplementary material for: The RNA-binding protein ROD1/PTBP3 cotranscriptionally defines AID-loading sites to mediate antibody class switch in mammalian genomes
Source: Cell Res. 2018 Aug 24;28(10):981–95. doi: 10.1038/s41422-018-0076-9 (PMC6170407; doi:10.1038/s41422-018-0076-9)
Supplement: Supplementary file 12 — Supplementary information, Figure S12 [file 41422_2018_76_MOESM12_ESM.pdf]

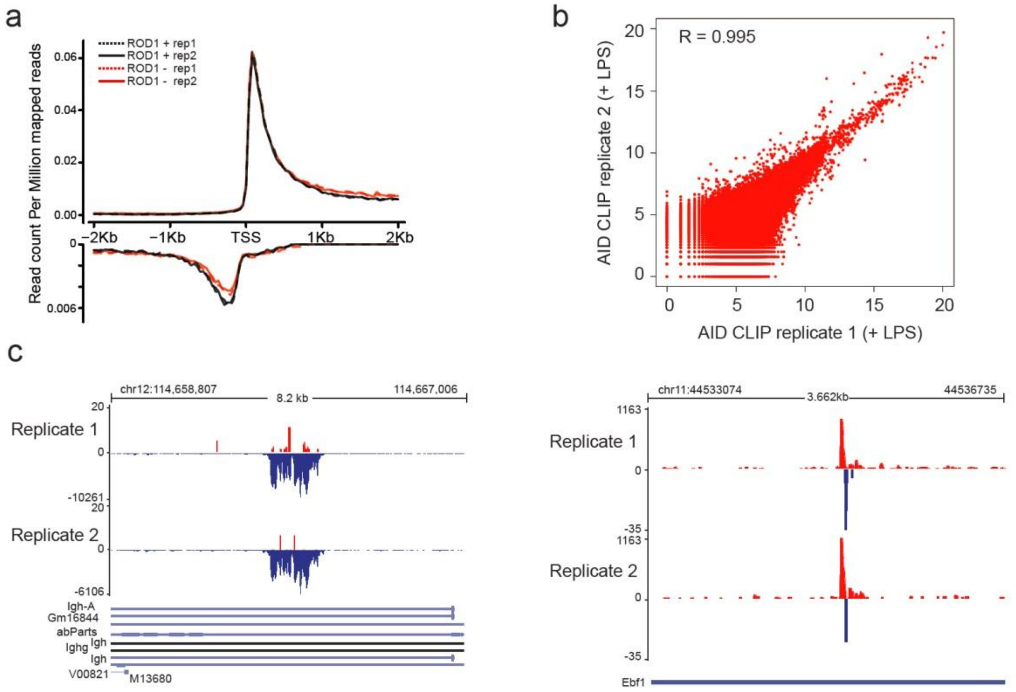

Supplementary Figure 12. Nascent RNA shows little change upon ROD1 depletion by Gro-seq. (**a**) The alignment of Gro-seq reads to TSSs in both the sense (upper) and antisense (lower) directions. The Gro-seq replicates in the presence of ROD1 are shown as black solid or dash lines. The Gro-seq replicates in the absence of ROD1 are shown as orange solid or dash lines. (**b**) The reproducibility of AID CLIP-seq analyzed by Pearson's correlation. Scatter plots show log_2-normalized read numbers between two replicates. R represents Pearson's correlation coefficient. (**c**) Snapshot of the AID CLIP-seq replicates at the *IgH* and *Ebf1* loci. Of note, AID binding sites at *Ebf1* were localized in a super-enhancer region[33]. The signal is shown in red or blue to indicate its localization on the Watson or Crick strand, respectively.
