# Supplementary material for: The RNA-binding protein ROD1/PTBP3 cotranscriptionally defines AID-loading sites to mediate antibody class switch in mammalian genomes
Source: Cell Res. 2018 Aug 24;28(10):981–95. doi: 10.1038/s41422-018-0076-9 (PMC6170407; doi:10.1038/s41422-018-0076-9)
Supplement: Supplementary file 13 — Supplementary information, Figure S13 [file 41422_2018_76_MOESM13_ESM.pdf]

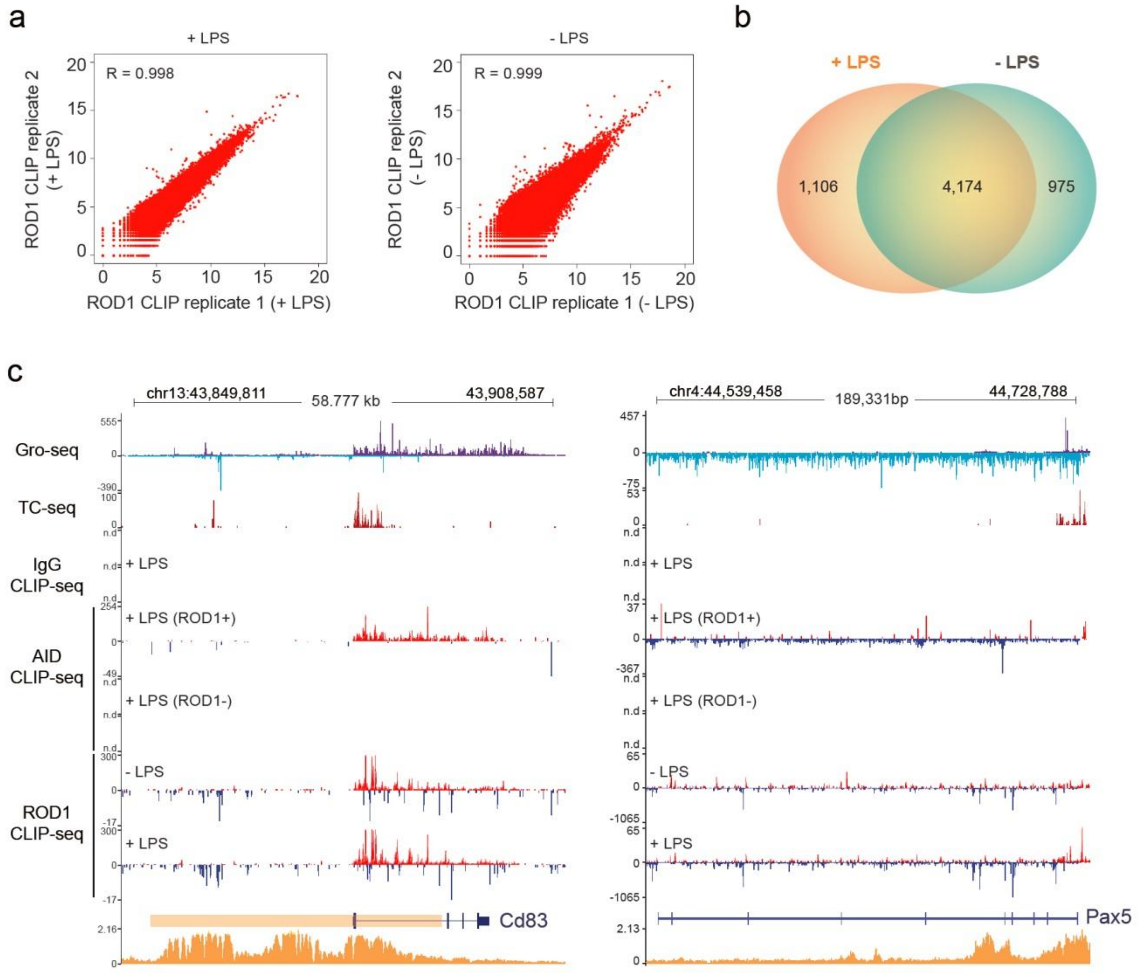

Supplementary Figure 13. ROD1 binding profiles are similar before and after LPS stimulation. (**a**) Pearson's correlation analysis of ROD1 CLIP-seq replicates. (**b**) Venn diagram of genes with ROD1 CLIP-seq peaks before and after LPS treatment. (**c**) Snapshot of the Gro-seq, TC-seq and CLIP-seq profiles in the vicinity of *Pax5* and *Cd83*. ROD1 and AID CLIP-seq signals are shown as red and blue for reads located on the Watson and Crick strands, respectively. Bottom: the H3K27Ac signal is shown in orange. The reported super-enhancer is marked with an orange box.
