# Supplementary material for: The RNA-binding protein ROD1/PTBP3 cotranscriptionally defines AID-loading sites to mediate antibody class switch in mammalian genomes
Source: Cell Res. 2018 Aug 24;28(10):981–95. doi: 10.1038/s41422-018-0076-9 (PMC6170407; doi:10.1038/s41422-018-0076-9)
Supplement: Supplementary file 14 — Supplementary information, Figure S14 [file 41422_2018_76_MOESM14_ESM.pdf]

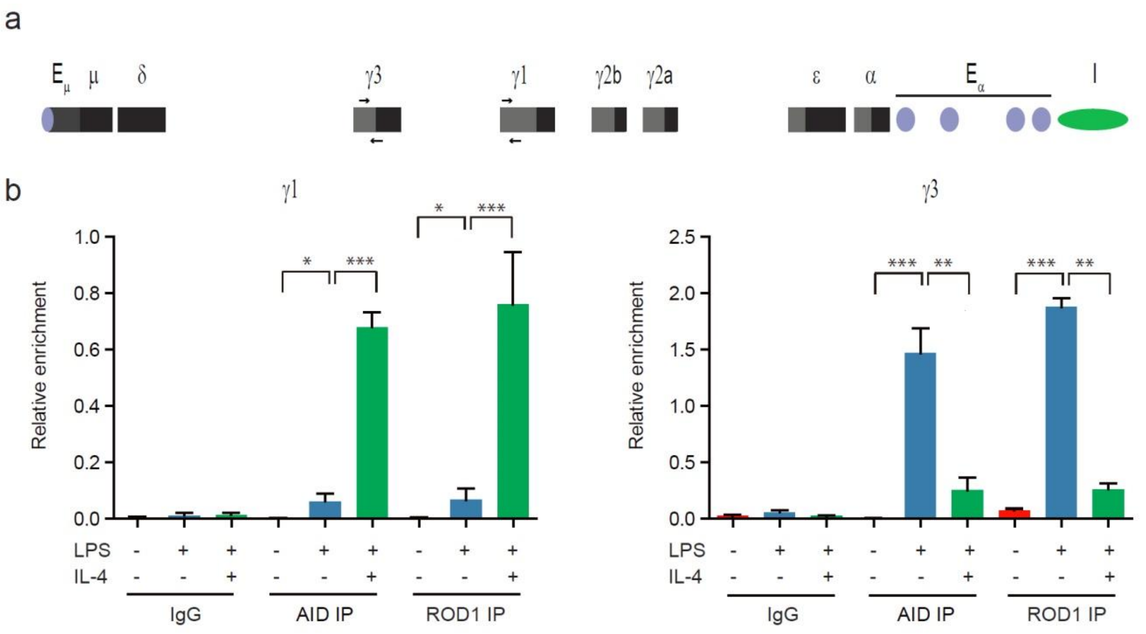

Supplementary Figure 14. ROD1 and AID occupy different γ-regions in response to different stimulations. (**a**) Diagram of the whole *IgH* locus. Primers targeting γ1 and γ3 are shown as arrowheads. (**b**) RIP-qPCR analysis of ROD1 and AID occupancy at γ1 and γ3 regions in B cells activated either by LPS or LPS plus IL-4 (n = 3). Data are shown as the mean ± SD (n = 3) and normalized against input. *P<0.05, **P<0.01, ***P<0.001, two-tailed Student's *t*-test.
