# Supplementary material for: The RNA-binding protein ROD1/PTBP3 cotranscriptionally defines AID-loading sites to mediate antibody class switch in mammalian genomes
Source: Cell Res. 2018 Aug 24;28(10):981–95. doi: 10.1038/s41422-018-0076-9 (PMC6170407; doi:10.1038/s41422-018-0076-9)
Supplement: Supplementary file 15 — Supplementary information, Figure S15 [file 41422_2018_76_MOESM15_ESM.pdf]

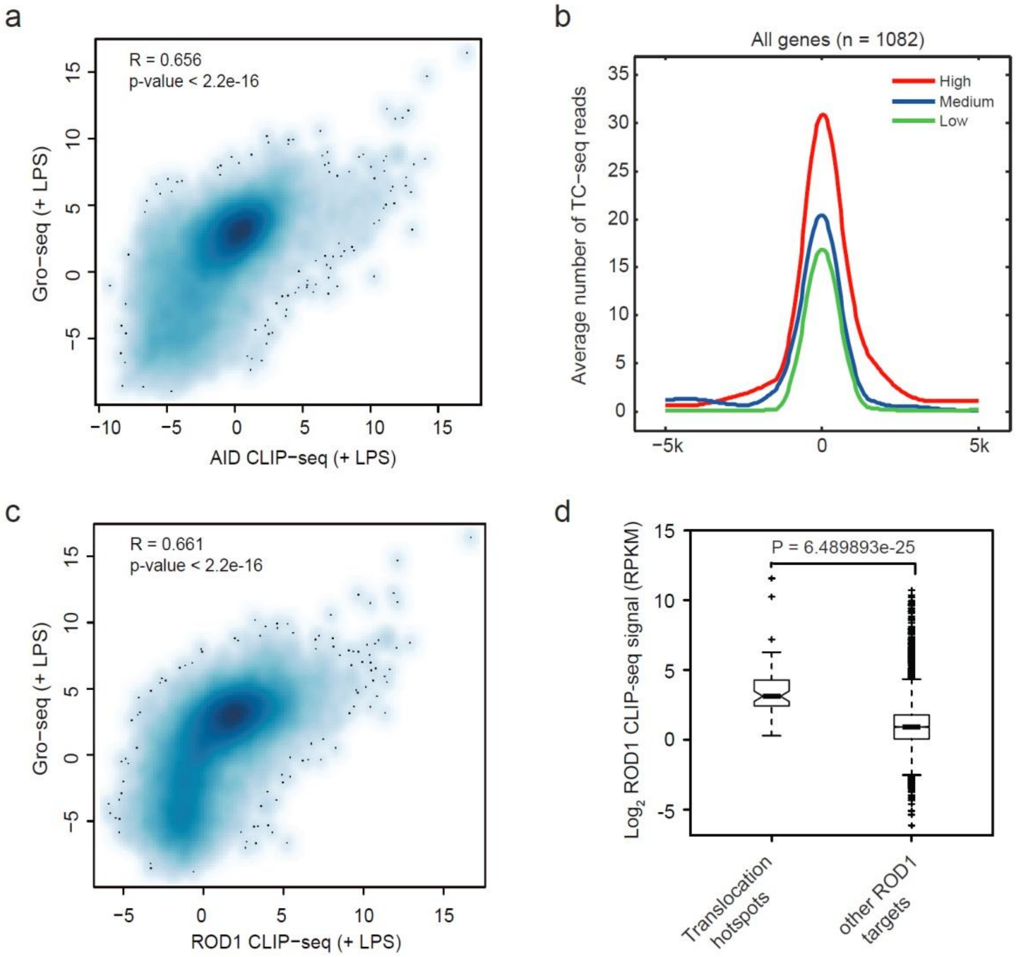

Supplementary Figure 15. ROD1 occupancy is highly correlated with TC-seq and Gro-seq signals. (**a**) The Pearson's correlation analysis of AID CLIP-seq to Gro-seq signals in LPS-activated B cells. Scatter plots represent the log_2-normalized RPKM values of Refseq genes between the two datasets. The *P* value and R are indicated. (**b**) ROD1 binding profiles around the center of the translocation hotspots detected by TC-seq. TC-seq-detected genes (n = 1082) were subgrouped into low, medium or high based on ROD1 CLIP-seq signal. (**c**) Pearson's correlation analysis of ROD1 CLIP-seq to Gro-seq signals in LPS-activated B cells. (**d**) Enrichment of ROD1 CLIP-seq reads among translocation hotspots in LPS-activated B cells. The comparison is performed between 61 translocation hotspot genes and 5,219 other ROD1 targets. *P* value was determined by Wilcoxon rank-sum test.
