# Supplementary material for: The RNA-binding protein ROD1/PTBP3 cotranscriptionally defines AID-loading sites to mediate antibody class switch in mammalian genomes
Source: Cell Res. 2018 Aug 24;28(10):981–95. doi: 10.1038/s41422-018-0076-9 (PMC6170407; doi:10.1038/s41422-018-0076-9)
Supplement: Supplementary file 16 — Supplementary information, Figure S16 [file 41422_2018_76_MOESM16_ESM.pdf]

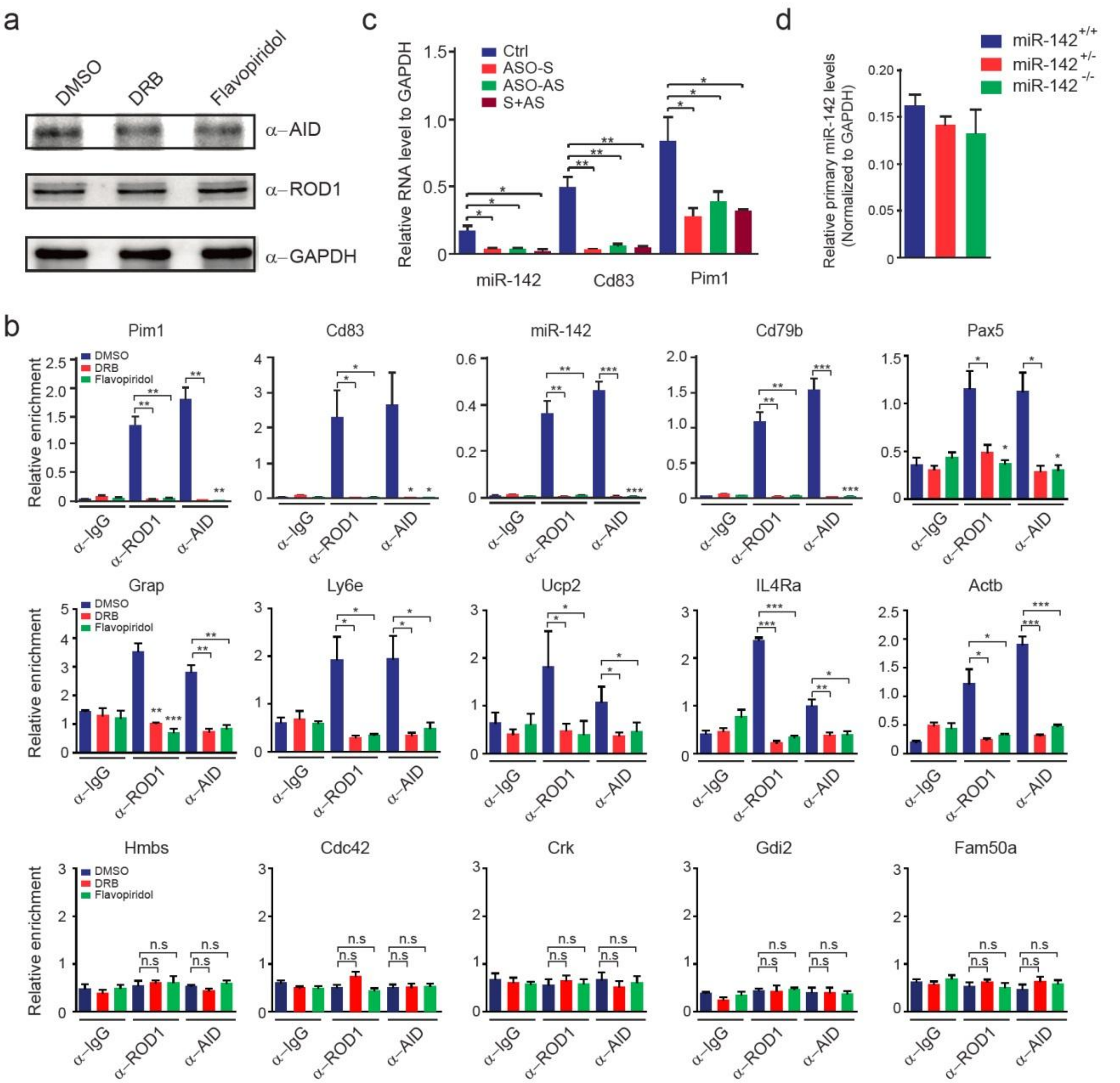

Supplementary Figure 16. The interaction of ROD1 with chromatin is dependent on transcription. (**a**) Western blot analysis of ROD1 and AID expression levels upon a transient block of transcription. GAPDH served as a loading control. (**b**) ChIP-qPCR for ROD1 occupancy at 10 AID targeted regions after blocking transcription. Five genes, namely, *Hmbs*, *Cdc42*, *Crk*, *Gdi2* and *Fam50a*, served as non-target controls. The relative enrichment was calculated by normalizing all of the data against input DNA. (**c**) RT-qPCR examination of bi-directional RNA upon ASO oligo treatment. The relative levels of bi-directional RNA were normalized to GAPDH. (**d**) RT-qPCR examination of primary miR-142 levels in different genotypes. Data are shown as the mean ± SD (n = 3) for (b), (**c**) and (d). *P<0.05, **P<0.01, ***P<0.001 and P>0.05 was non-significant (n.s) as determined by two-tailed Student's t-test.
