# Supplementary material for: The RNA-binding protein ROD1/PTBP3 cotranscriptionally defines AID-loading sites to mediate antibody class switch in mammalian genomes
Source: Cell Res. 2018 Aug 24;28(10):981–95. doi: 10.1038/s41422-018-0076-9 (PMC6170407; doi:10.1038/s41422-018-0076-9)
Supplement: Supplementary file 17 — Supplementary information, Figure S17 [file 41422_2018_76_MOESM17_ESM.pdf]

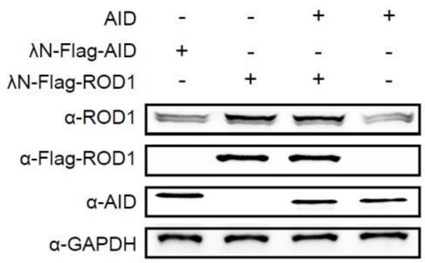

Supplementary Figure 17. The expression levels of AID and ROD1 in transfected HEK293 cells by Western blotting. GAPDH served as a loading control, Related to Fig. 6e.
