# Supplementary material for: The RNA-binding protein ROD1/PTBP3 cotranscriptionally defines AID-loading sites to mediate antibody class switch in mammalian genomes
Source: Cell Res. 2018 Aug 24;28(10):981–95. doi: 10.1038/s41422-018-0076-9 (PMC6170407; doi:10.1038/s41422-018-0076-9)
Supplement: Supplementary file 18 — Supplementary information, Figure S18 [file 41422_2018_76_MOESM18_ESM.pdf]

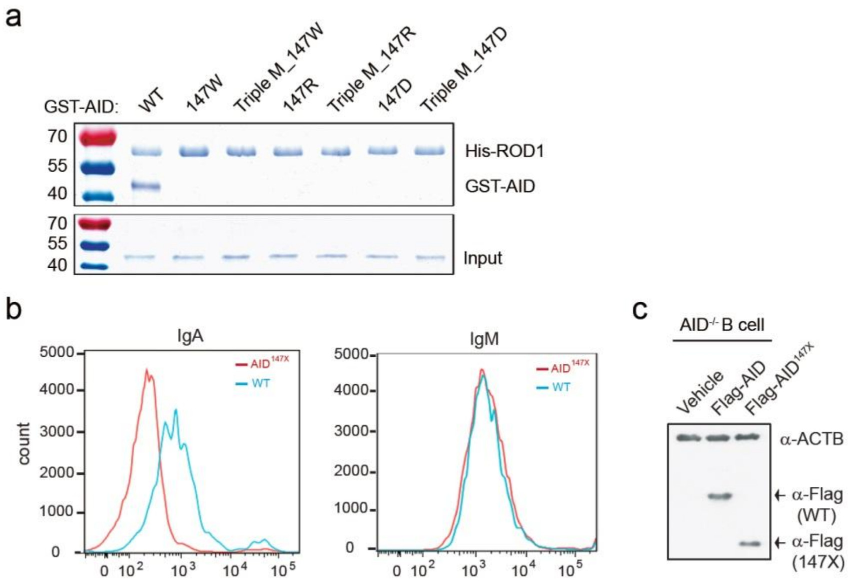

Supplementary Figure 18. The 147X mutation disrupts the interaction of AID with ROD1 *in vitro*. (**a**) His-ROD1 pulls down GST-AID but not its 147X mutation variant. Triple M stands for combined mutations at residues 139, 147 and 151. (**b**) CSR to IgA is compromised in AID^{147X} knock-in CH12F3 cells after stimulation with CIT (CD40L, IL-4 and TGF-β). (**c**) The expression levels of Flag-tagged AID and AID^{147X} mutant in retrovirally transduced $AID^{-/-}$ B cells. The two arrow heads mark the positions of WT and 147X mutant proteins, respectively.
