# Supplementary material for: The RNA-binding protein ROD1/PTBP3 cotranscriptionally defines AID-loading sites to mediate antibody class switch in mammalian genomes
Source: Cell Res. 2018 Aug 24;28(10):981–95. doi: 10.1038/s41422-018-0076-9 (PMC6170407; doi:10.1038/s41422-018-0076-9)
Supplement: Supplementary file 19 — Supplementary information, Figure S19 [file 41422_2018_76_MOESM19_ESM.pdf]

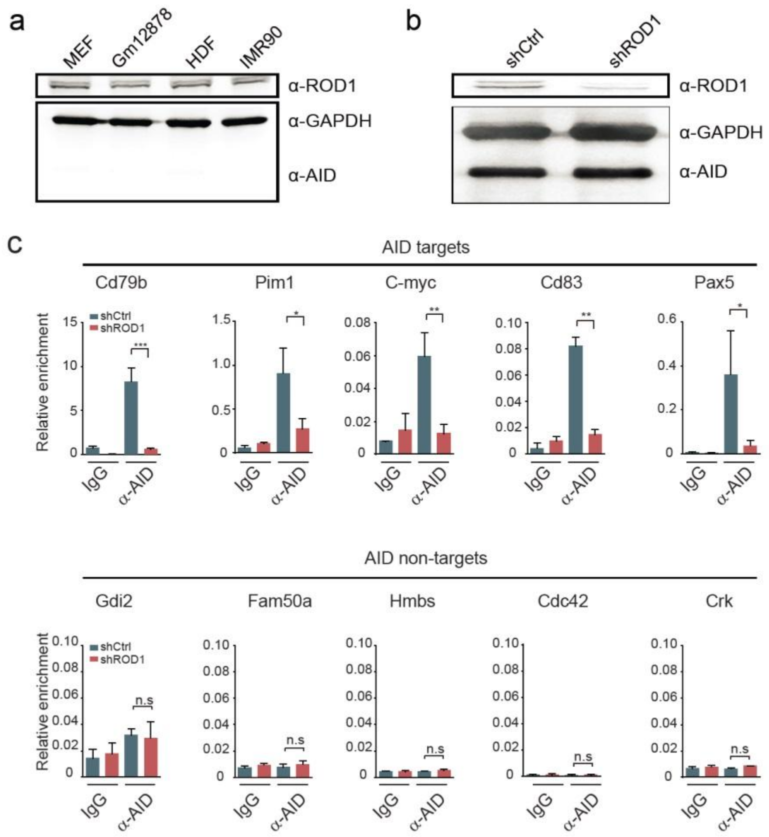

Supplementary Figure 19. ROD1 determines AID targeting in human Ramos B cells. (**a**) ROD1 and AID expression levels in diverse mouse and human cell lines. GAPDH served as a loading control. AID is not expressed in the examined cells. (**b**) Western blotting to examine ROD1 knockdown effects in Ramos B cells. (**c**) ChIP-qPCR for AID occupancy in five AID-targeted regions upon ROD1 depletion in Ramos cells. ChIP was performed with anti-AID or control IgG. Five genes, namely *Hmbs, Cdc42, Crk, Gdi2* and *Fam50a*, served as non-target controls. Data are shown as the mean ± SD (n = 3) and normalized against input DNA. *P<0.05, **P<0.01, ***P<0.001 and P>0.05 was non-significant (n.s) as determined by two-tailed Student's *t*-test.
