# Supplementary material for: The RNA-binding protein ROD1/PTBP3 cotranscriptionally defines AID-loading sites to mediate antibody class switch in mammalian genomes
Source: Cell Res. 2018 Aug 24;28(10):981–95. doi: 10.1038/s41422-018-0076-9 (PMC6170407; doi:10.1038/s41422-018-0076-9)
Supplement: Supplementary file 20 — Supplementary information, Table S1 [file 41422_2018_76_MOESM20_ESM.docx]

Supplementary Table 1. List of unique AID partners identified by IP-MS.

| **Accession** | **Gene name (AID + LPS)** | **Gene name (AID - LPS)** |
| --- | --- | --- |
| B0V2N8 | Anxa2 | Anxa2 |
| E9Q223 | Hbb-bs | Hbb-bs |
| E9Q5F6 | Ubc | Ubc |
| F8WIX8 | Hist1h2al | Hist1h2al |
| H3BLM8 | Gbf1 | Gbf1 |
| P03930 | Mtatp8 | Mtatp8 |
| P05202 | Got2 | Got2 |
| P08113 | Hsp90b1 | Hsp90b1 |
| P11247 | Mpo | Mpo |
| P20029 | Hspa5 | Hspa5 |
| P42125 | Eci1 | Eci1 |
| P47878 | Igfbp3 | Igfbp3 |
| P51881 | Slc25a5 | Slc25a5 |
| P56480 | Atp5b | Atp5b |
| P60710 | Actb | Actb |
| P62806 | Hist1h4a | Hist1h4a |
| Q8VEM8 | Slc25a3 | Slc25a3 |
| Q99LC5 | Etfa | Etfa |
| Q9CPN9 | 2210010C04Rik | 2210010C04Rik |
| Q9DB77 | Uqcrc2 | Uqcrc2 |
| Q9JKR6 | Hyou1 | Hyou1 |
| A0A0G2JE84 | Tmigd3 |  |
| A0A0N4SV66 | H2afj |  |
| A2AIM5 | Tpm2 |  |
| B1ARR7 | Eno1 |  |
| B1ASE2 | Atp5h |  |
| B1AWZ5 | Nipsnap3b |  |
| B1AXW5 | Prdx1 |  |
| D3YUK4 | Ndufb10 |  |
| E0CX78 | Fcrla |  |
| E0CZ27 | H3f3a |  |
| F7C545 | Ankhd1 |  |
| F7D3P8 | Atp5o |  |
| F8WIT2 | Anxa6 |  |
| G3UX26 | Vdac2 |  |
| G3UZ01 | Rod1/Ptbp3 |  |
| G3UZ67 | Paip1 |  |
| G3UZL4 | Rab11b |  |
| G3UZZ2 | Tapbp |  |
| J3QP71 | Bsg |  |
| M0QWA7 | Itgb2 |  |
| M0QWX7 | Cox4i1 |  |
| O08692 | Ngp |  |
| O08749 | Dld |  |
| O35129 | Phb2 |  |
| O35744 | Chil3 |  |
| P00405 | Mtco2 |  |
| P01831 | Thy1 |  |
| P01896 | H-2 class I histocompatibility  antigen, alpha chain (Fragment) OS=Mus musculus PE=2 SV=1 - [HA1Z_MOUSE] |  |
| P01942 | Hba |  |
| P04441-2 | Isoform Short of H-2 class II histocompatibility antigen gamma chain OS=Mus musculus GN=Cd74 - [HG2A_MOUSE] |  |
| P05555-2 | Isoform 2 of Integrin alpha-M OS=Mus musculus GN=Itgam - [ITAM_MOUSE] |  |
| P06346 | H2-Ab1 |  |
| P07724 | Alb |  |
| P08003 | Pdia4 |  |
| P08249 | Mdh2 |  |
| P09103 | P4hb |  |
| P09671 | Sod2 |  |
| P10107 | Anxa1 |  |
| P10853 | Hist1h2bf |  |
| P11911 | Cd79a |  |
| P12787 | Cox5a |  |
| P14211 | Calr |  |
| P14438 | H2-Aa |  |
| P15379-2 | Isoform 13 of CD44 antigen OS=Mus musculus GN=Cd44 - [CD44_MOUSE] |  |
| P15530 | Cd79b |  |
| P15864 | Hist1h1c |  |
| P19437 | Ms4a1 |  |
| P20108 | Prdx3 |  |
| P20152 | Vim |  |
| P24668 | M6pr |  |
| P27773 | Pdia3 |  |
| P28078 | H2-DMa |  |
| P28843 | Dpp4 |  |
| P29391 | Ftl1 |  |
| P29477 | Nos2 |  |
| P31725 | S100a9 |  |
| P48036 | Anxa5 |  |
| P51437 | Camp |  |
| P61027 | Rab10 |  |
| P63038 | Hspd1 |  |
| P67778 | Phb |  |
| P68368 | Tuba4a |  |
| Q03265 | Atp5a1 |  |
| Q31099 | H2-DMb2 |  |
| Q3U7R1-2 | Isoform 2 of Extended synaptotagmin-1 OS=Mus musculus GN=Esyt1 - [ESYT1_MOUSE] |  |
| Q3UR42 | Dhx9 |  |
| Q3UV17 | Krt76 |  |
| Q504P4 | Hspa8 |  |
| Q5SX48 | Slc25a11 |  |
| Q60605 | Myl6 |  |
| Q60931 | Vdac3 |  |
| Q60932-2 | Isoform Mt-VDAC1 of Voltage-dependent anion-selective channel protein 1 OS=Mus musculus GN=Vdac1 - [VDAC1_MOUSE] |  |
| Q61425 | Hadh |  |
| Q61735 | Cd47 |  |
| Q62351 | Tfrc |  |
| Q64433 | Hspe1 |  |
| Q8BH59 | Slc25a12 |  |
| Q8BMK4 | Ckap4 |  |
| Q8CDA1-3 | Isoform 3 of Phosphatidylinositide phosphatase SAC2 OS=Mus musculus GN=Inpp5f - [SAC2_MOUSE] |  |
| Q8K327 | Champ1 |  |
| Q8VDD5 | Myh9 |  |
| Q922R8 | Pdia6 |  |
| Q99KF1 | Tmed9 |  |
| Q9CQQ7 | Atp5f1 |  |
| Q9CZ13 | Uqcrc1 |  |
| Q9D1D4 | Tmed10 |  |
| Q9D2G2-2 | Isoform 2 of Dihydrolipoyllysine-residue succinyltransferase component of 2-oxoglutarate dehydrogenase complex, mitochondrial OS=Mus musculus GN=Dlst - [ODO2_MOUSE] |  |
| Q9DC69 | Ndufa9 |  |
| Q9ERN0 | Scamp2 |  |
| Q9WVE0 | Aicda |  |
| Q9R1Q7 | Plp2 |  |
| S4R1M0 | Ptprc |  |
| S4R257 | Gapdh |  |
| A0A0G2JGY4 |  | Mdh2 |
| A2A6Z7 |  | Ccdc73 |
| A2AW79 |  | Dock7 |
| D3Z6F5 |  | Atp5a1 |
| F6Y0Q7 |  | Rbm33 |
| G3X9D5 |  | Hist2h2bb |
| O88544 |  | Cops4 |
| O88974-2 |  | Isoform 2 of Histone-lysine N-methyltransferase SETDB1 OS=Mus musculus GN=Setdb1 - [SETB1_MOUSE] |
| P51942 |  | Matn1 |
| P52480 |  | Pkm |
| P97868-2 |  | Isoform 2 of E3 ubiquitin-protein ligase RBBP6 OS=Mus musculus GN=Rbbp6 - [RBBP6_MOUSE] |
| Q3UFB7 |  | Ntrk1 |
| Q5SQG5 |  | Phb |
| Q61781 |  | Krt14 |
| Q6PFQ7-2 |  | Isoform 2 of Ras GTPase-activating protein 4 OS=Mus musculus GN=Rasa4 - [RASL2_MOUSE] |
| Q792Z1 |  | Try10 |
| Q8BFZ3 |  | Actbl2 |
| Q8R2S8 |  | Cd177 |
| Q9JKK8 |  | Atr |
| Q9Z0K8 |  | Vnn1 |
