# Supplementary material for: The RNA-binding protein ROD1/PTBP3 cotranscriptionally defines AID-loading sites to mediate antibody class switch in mammalian genomes
Source: Cell Res. 2018 Aug 24;28(10):981–95. doi: 10.1038/s41422-018-0076-9 (PMC6170407; doi:10.1038/s41422-018-0076-9)
Supplement: Supplementary file 3 — Supplementary information, Figure S3 [file 41422_2018_76_MOESM3_ESM.pdf]

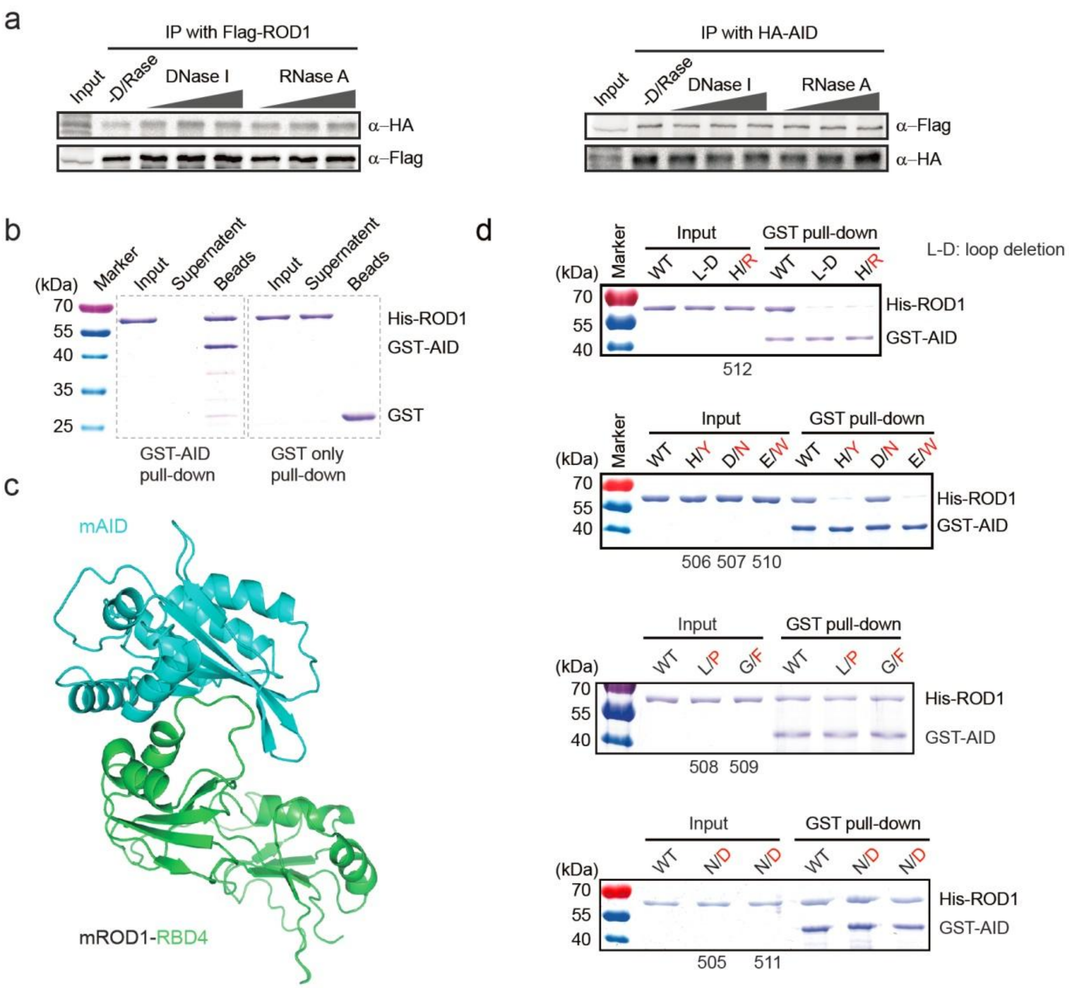

Supplementary Figure 3. Key residues contribute to direct interactions between ROD1 and AID. (**a**) ROD1 interacts with AID in a co-IP assay. HEK293 cells were co-transfected with Flag-tagged ROD1 and HA-tagged AID. The co-IP was performed with anti-Flag and anti-HA antibodies, the immunoprecipitates were analyzed by Western blot with antibody to HA or Flag. Three different concentrations of DNase I (0.1 U/μl, 0.01 U/μl and 0.001 U/μl) and RNase A (1 μg/μl, 0.1 μg/μl and 0.01 μg/μl) were used. (**b**) The direct interaction between ROD1 and AID by GST pull-down assay. (**c**) The 3D interacting complex of AID and ROD1 modeled by PRISM. mAID is shown in cyan, whereas the ROD1 RBD4 domain is in green. (**d**) GST-AID pulled-down proteins from the inputs of bacterially expressed His-ROD1-mutated variants.
